# Isolation and characterization of three novel lytic phages against K54 serotype carbapenem-resistant hypervirulent *Klebsiella pneumoniae*

**DOI:** 10.3389/fcimb.2023.1265011

**Published:** 2023-12-12

**Authors:** Chengju Fang, Xiaoyi Dai, Li Xiang, Yichuan Qiu, Ming Yin, Yu Fu, Ying Li, Luhua Zhang

**Affiliations:** The School of Basic Medical Science and Public Center of Experimental Technology, Southwest Medical University, Luzhou, Sichuan, China

**Keywords:** CR-hvKp, phage, cocktail therapy, lytic phage, antibacterial activity

## Abstract

The emergence of carbapenem-resistant hypervirulent *Klebsiella pneumoniae* (CR-hvKP) has driven us to explore alternative treatments for the limitation of antimicrobial agents. Lytic phages are considered a promising alternative treatment for CR-hvKP infection. In this study, we reported three novel lytic phages, vB_KpnA_SCNJ1-Z, vB_KpnS_SCNJ1-C, and vB_KpnM_SCNJ1-Y, against a CR-hvKP strain SCNJ1, and they possess genomes of double-stranded DNA with a size of 43,428 bp, 46,039 bp, and 50,360 bp, respectively. Phylogenetic analysis demonstrated that vB_KpnA_SCNJ1-Z belongs to the family *Autographiviridae* within the class *Caudoviricetes*, while vB_KpnS_SCNJ1-C and vB_KpnM_SCNJ1-Y are unclassified *Caudoviricetes*. The phages showed a narrow host range only lysing 1 of 50 tested clinical bacterial strains. The one-step growth curves and stability results showed that the phages displayed relatively short latency periods, with broad pH (pH 3-14) and thermal stabilities (20–60°C). The phages showed significant inhibition of the biofilm formation by SCNJ1 and strong antibacterial activity *in vitro*. In the mouse model, we demonstrated that administration of a single phage or phage cocktail significantly reduced bacteria loads in the lung, liver, and spleen, and effectively rescued mice from the infection of the SCNJ1 strain, with a survival rate of 70-80%. These findings suggested the three phages have great potential as an alternative therapy with favorable stability and strong antibacterial activity both *in vivo* and *in vitro* for the treatment of CR-hvKP infection.

## 1 Introduction

Hypervirulent *Klebsiella pneumoniae* (hvKp) is an evolving pathotype of *Klebsiella pneumoniae* (*K*. *pneumonia*) with hypermucoviscosity and hypervirulence causing community-associated and hospital-associated infections globally, including central nervous system infection, endophthalmitis, pyogenic liver abscess, pneumonia, and meningitis (Russo and Marr, 2019; Serban et al., 2021; Zhu et al., 2021). Carbapenem-resistant hypervirulent *Klebsiella pneumoniae* (CR-hvKP) is a clinically significant pathogen known for its capacity to cause severe and life-threatening infections. The prevalence of CR-hvKP has been steadily increasing since 2010, with spread dissemination observed. ST23, ST11, and K1, K2 are the predominant ST types and serotypes of CR-hvKP in China, respectively (Lan et al., 2021). In 2016, a fatal outbreak of ventilator-associated pneumonia caused by CR-hvKP occurred in a Chinese hospital’s intensive care unit (ICU). Despite antibiotic treatment, all patients experienced poor responses and ultimately succumbed to severe lung infection, multiorgan failure, or septic shock (Gu et al., 2018). In a Chinese teaching hospital, 11 patients were infected with CR-hvKP, resulting in 3 fatalities due to severe CR-hvKP infection. All 11 CR-hvKP isolates displayed high-level resistance to commonly used antibiotics, except for susceptibility to colistin, tigecycline, and ceftazidime/avibactam, which are the last resort therapeutic options against CR-hvKP infection (Yu et al., 2018; Zhu et al., 2022). Recent occurrences of colistin- or tigecycline-resistant CR-hvKP clinical isolates highlight the urgent need to develop alternative clinical treatments as substitutes for conventional antimicrobial drugs (Li et al., 2019; Zhang et al., 2021b; Zhang et al., 2021c).

Phage therapy is widely regarded as a promising alternative to conventional antibiotics due to its ability to lyse host bacterial cells. Briefly, the infection process commences as the phage attaches to the host cell surface *via* a specific receptor. Successful binding triggers conformational changes in the phage’s baseplate, causing sheath shrinkage and subsequent injection of the phage’s nucleic acid into the host cell. The progeny phages synthesized within the bacterial cell are obligated to induce host cell lysis, thereby liberating the virions into the surrounding environment (Stone et al., 2019). Multiple *in vivo* and *in vitro* studies have shown that phage therapy can be safely administered topically, orally, inhaled, and intravenously. Several studies have utilized animal models, such as mice or larvae, to investigate the therapeutic potential of phages against *K. pneumoniae*-induced pneumonia, liver abscesses, and burn infections (Lin et al., 2014; Chadha et al., 2017; Anand et al., 2020). Importantly, phage therapy has been implemented in clinical treatment for the management of severe infections caused by multi-drug-resistant *K. pneumoniae* (Cano et al., 2021).

However, research referring to phage therapy for the treatment of CR-hvKP infections was almost blank, including serotype K54 CR-hvKP infections. In this study, we isolated three novel phages against a CR-hvKP clinical strain with a K54 serotype from sewage and river water. We examined their biological characteristics and genetic backgrounds and investigated their therapeutic potential using the mouse infection model.

## 2 Materials and methods

### 2.1 Bacterial strain, phage isolation, and identification

For phage isolation, a ST29 CR-hvKP clinical strain with K54 serotype, SCNJ1 (Yuan et al., 2019), which was recovered from the sputum of a patient with acute bronchiolitis in a hospital in Sichuan Province in November 2018, was employed as the host strain. vB_KpnA_SCNJ1-Z, vB_KpnS_SCNJ1-C, and vB_KpnM_SCNJ1-Y were isolated from the sewage water of the Southwest Medical University’s Affiliated Hospital for Traditional Chinese Medicine, Yangtze River, and Yudai River in Luzhou, Sichuan, China, respectively. 100 mL of sewage or river water was centrifuged for 20 min at 12,000 rpm, 4°C to remove debris, and a 0.22-μm filter (JETBIOFIL, China) was used to filter the supernatant. To enrich phage, 30 mL filtered supernatant with 15 mL 3×LB broth was inoculated with 200 µL exponential host cells and cultured for 12 h at 37°C with shaking at 220 rpm. The mixture was centrifuged at 12,000 rpm, 4°C for 10 min, and the supernatant was collected to isolate the phage using the double-layer agar method (Adams, 1959). Single plaques were purified in 5 passages until the formation of uniform plaques was obtained. The plaque-purified phages were stored in SM buffer at -80°C for further experiments.

### 2.2 Transmission electron microscopy

The TEM method was performed as previously described by Taruna Anand et al. with slight variations (Anand et al., 2020). Briefly, the purified phages were placed on carbon-coated copper grids, allowed to stand for 5 min, and stained with 2% phosphotungstic acid for 30 seconds. The morphology of the phages was observed under a Hitachi transmission electron microscope HT7820 (Japan) at 80 kV.

### 2.3 Optimal multiplicity of infection and one-step growth curve

The method of optimal multiplicity of infection is similar to those described by Mingfang Pu et al. with slight changes (Pu et al., 2022). Briefly, exponential host cells were mixed with the phages with different multiplicities of infection (MOI: 0.0001,0.001,0.01,0.1,1,10,100), then shaken for 12 h at 37°C (220 rpm). The phage titers were determined using the double-layer agar method. The proportion with the highest phage titer was the optimal multiplicity of infection. One-step curves were performed as previously described (Hyman and Abedon, 2009). Briefly, 1mL of phage (1×10^8^ PFU) was mixed with 9 mL of the exponential host cells (1×10^9^ CFU) at the MOI of 0.1. The mixture was incubated at 37°C with shaking (150 rpm), 5 min after mixing phages and bacteria, the mixture was 1000 fold diluted in LB medium to lower the bacterial concentration and avoid reinfection. The diluted mixture was continued to shake and 100 μL sample was collected at 2-min intervals during 30 min for titration. The assay was performed in triplicate.

### 2.4 Host range determination

The method of host range determination was performed as previously described by Yuting Shang et al. with slight variations (Shang et al., 2021). To investigate the host range of the phages, spot-test assays (Tan et al., 2020) were performed on 50 clinical strains (**Supplementary Table S1**). Briefly, 5 µL filtered phage suspension (~10^10^ PFU/mL) was dropped onto plates that were covered with different bacterial lawns. The plates were incubated at 37°C for 24 h to observe plaque formation.

### 2.5 pH and thermal stabilities

The methods of pH and thermal stabilities were performed as previously described by Narges Torkashvand et al. with slight changes (Torkashvand et al., 2023). In brief, for the pH stability test, 100 µL phage (~10^10^ PFU/mL) was added to 900 µL SM buffer of different pH (1-14) and incubated for 1 h at 37°C. For the thermal stability test, 100 µL phage suspension (~10^10^ PFU/mL) was mixed with 900 µL SM buffer, and incubated for 1 h at temperatures ranging from 20 to 80°C. The phage titers were determined by the double-layer agar method. All assays were performed in triplicate.

### 2.6 Chloroform susceptibility test

To determine whether there are lipids in the viral capsid, the sensitivities of the phages to chloroform were examined. The method of chloroform susceptibility test was performed as previously described by Yubing Chen et al. with slight changes (Chen et al., 2023). In brief, 1 mL phage suspension was mixed with 100 µL chloroform, the mixture was vigorously shaken for 1 min and then incubated at room temperature for 30 min. The mixture was centrifuged at 10,000 rpm for 10 min and the supernatant was collected and plated for phage titer using the double-layer agar method.

### 2.7 Antibiofilm activity of phages

The inhibitory effects of the phages on biofilm formation were evaluated as previously described with slight modifications (Mulani et al., 2022). Overnight grown host cells were diluted 1:100 with LB broth to approximately 10^7^ CFU/mL and mixed with phages with different MOI (100, 10, 1, and 0.1). 200 µL of the mixture, 200 µL of LB as negative controls, and 200 µL of bacterial suspensions as positive controls were added individually in triplicate into a sterile 96-well microplate. The 96-well microplate was incubated for 24 h at 37°C, then, washed three times with sterile phosphate-buffered saline (PBS). After 15 min of methanol fixation, all wells were added with 200 µL of 0.1% ammonium oxalate crystal violet solution, stained for 20 min, washed three times with ultrapure water, dried, and solubilized with 200 µL of 33% acetic acid for 10 min. Optical density was determined at 595 nm using a microplate reader.

### 2.8 Antibacterial activity *in vitro*

The method of antibacterial activity *in vitro* was performed as previously described by Jingyun Fu et al. with slight changes (Fu et al., 2023). Briefly, the host cells were cultured to exponential phase, diluted to 10^7^ CFU/mL (10 mL), and infected with phages (10 mL) with an MOI of 1, 10, and 100. The mixture was incubated at 37°C, 150 rpm. For the control group, 10 mL LB and 10 mL bacteria (10^7^ CFU/mL) were mixed and cultured under the same conditions. 100 µL of each mixture was removed at 0, 1, 2, 3, 4, 5, and 6 h, diluted, and plated on LB plated to count CFU per milliliter. Each experiment was carried out in triplicates.

### 2.9 DNA extraction

The method of DNA extraction was performed as previously described by Na Li et al. with slight changes (Li et al., 2021). In brief, filtered phages (16 mL) were incubated for 1 h at 37°C with 10 µL RNaseA (25 mg/mL) and 20 µL DNaseI (1 U/µL) to remove host bacterial nucleic acid, followed by the addition of the 10% (w/v) polyethylene glycol 8000 (PEG8000) and sodium chloride (NaCl) solution. The mixture was precipitated at 4°C overnight and centrifuged at 11,000 rpm for 30 min at 4°C. The pellet which contained phage particles was resuspended with 1 mL of SM buffer and mixed with an equal volume chloroform to remove the proteinaceous material. The process was repeated twice and the aqueous phase was used to extract phage DNA with the omega BIO-TEK Viral DNA Kit D3892 (omega, Norcross, GA, USA) following the manufacturer’s protocol.

### 2.10 Sequencing and analysis of phage genomes

The genomic DNA of three phages was sequenced by Shanghai Majorbio Bio-Pharm Technology Co., Ltd. (Shanghai, China) using the HiSeq 2000 (Illumina, San Diego, CA, USA) Sequencer. The raw sequenced reads were quality controlled and trimmed with FastQC 0.11.2 (https://www.bioinformatics.babraham.ac.uk/projects/fastqc/) and Trimmomatic 0.36 (http://www.usadellab.org/cms/?page=trimmomatic), respectively. The high-quality reads were assembled into a single raw contig *via* SPAdes 3.5.0. (http://cab.spbu.ru/software/spades/). Genes were predicted and annotated using Rapid Annotation using Subsystem Technology (RAST, http://rast.nmpdr.org/). Circular genome maps of the phage genomes were drawn using CGView (https://paulstothard.github.io/cgview/). The antimicrobial resistance genes (AMRs) and the virulence genes were screened in the Comprehensive Antibiotic Resistance Database (CARD) (https://card.mcmaster.ca/analyze) and the VirulenceFinder (https://cge.food.dtu.dk/services/VirulenceFinder/), respectively. Phylogenetic trees based on large terminase and major capsid protein were conducted by the Neighbor-joining method using MEGA11.0 with 1000 bootstrap replications. The complete genome sequence of the three phages was aligned with other phages using the BLASTn tool in the NCBI database, and complete genome sequence similarity between the three phages and other phages was visualized using the Circoletto program (http://bat.ina.certh.gr/tools/circoletto/).

### 2.11 Mouse experiments

The method of mouse experiments was performed as previously described by Mingfang Pu et al. with slight changes (Pu et al., 2022). Briefly, 91 female specific-pathogen-free (SPF) BALB/c mice, aged 4-5 weeks and weighed 18-20 g, were purchased from Beijing Huafukang Biotechnology Co., Ltd. All animal experiments were followed National Guidelines for Experimental Animal Welfare (Ministry of Science and Technology of China) and approved by the Animal Welfare and Research Ethics Committee at Southwest Medical University (swmu20230036). To construct immunodeficient mouse models, all mice were given cyclophosphamide (LKT labs, America) at a dose of 300 mg/kg for three days before the experiment.

Thirteen mice were randomly assigned to each group and anesthetized by inhalation of isoflurane. Four experimental groups and the positive control group were infected with 50 µL (1 × 10^6^ CFU/each) of SCNJ1 strain via nasal drip, two hours after infection, 50 µL of phages (1 × 10^8^ PFU/each) of vB_KpnA_SCNJ1-Z, vB_KpnS_SCNJ1-C, vB_KpnM_SCNJ1-Y, a cocktail (vB_KpnA_SCNJ1-Z, vB_KpnS_SCNJ1-C, and vB_KpnM_SCNJ1-Y in equal proportion) or 50 µL PBS was administered *via* nasal drip, respectively. The negative group and the safe test group were given 50 µL PBS first and treated with either the 50 µL of PBS or the cocktail after 2 hours. The mice were observed for survival curves every 24 h for seven days. 30 hours after phage treatment, three mice were randomly selected from each group for dissection. The left lung, liver, and spleen were removed under sterile conditions, and homogenized, and the bacterial load of each organ was determined using the bacterial dilution plate method. The right lung was fixed in 10% formalin, embedded, sectioned, and stained with hematoxylin and eosin (H&E) to observe pathological changes.

### 2.12 Statistical analysis

All data were analyzed using GraphPad Prism 9.4.0 (GraphPad Software, Inc., San Diego, CA, USA) and expressed as means and standard deviation values. Survival curve analyses were performed using Kaplan–Meier survival analysis with the log-rank test. Student’s t-test was utilized to compare two groups and one-way analysis of variance (ANOVA) was used to compare multiple groups. Statistical significance was set at p<0.05.

## 3 Results

### 3.1 Isolation and morphology of phages

**Figure 1:**
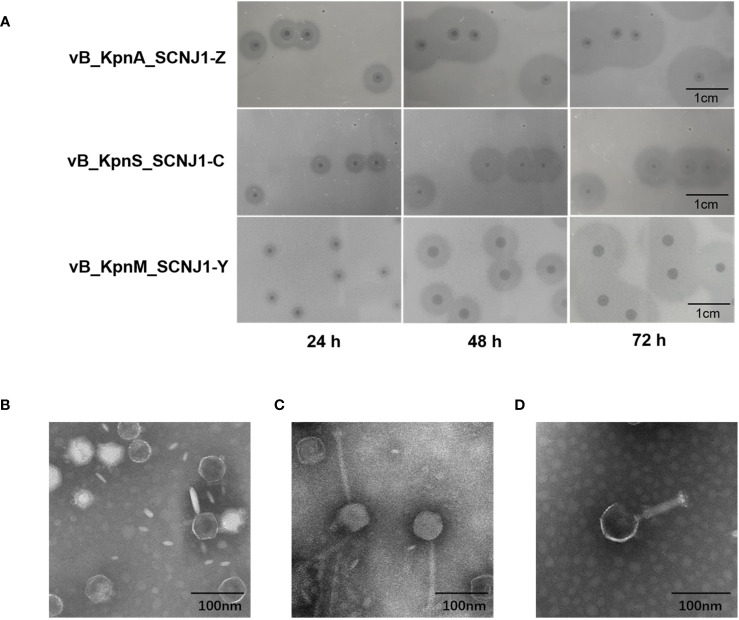
Plaques and Transmission electron micrograph image of *Klebsiella* phages. **(A)** Phage plaques on the lawn of *K*. *pneumoniae* SCNJ1. Transmission electron micrograph image of *Klebsiella* phages **(B)** vB_KpnA_SCNJ1-Z, **(C)** vB_KpnS_SCNJ1-C, and **(D)** vB_KpnM_SCNJ1-Y.

Three novel lytic phages, named vB_KpnA_SCNJ1-Z, vB_KpnS_SCNJ1-C, and vB_KpnM_SCNJ1-Y, formed circular translucent plaques on lawns of *K. pneumoniae* SCNJ1 with haloes distributed around the plaque center. The plaque images taken every 24 h for 3 days revealed the haloes expanding in size with time and indicated the presence of depolymerase which can digest bacterial capsules (Adams and Park, 1956) (**Figure 1A**). The transmission electron microscopy (TEM) showed that vB_KpnA_SCNJ1-Z had an icosahedral head, with a head diameter of 64.8 ± 2.0 nm and a tail length was 12 ± 2.5 nm (**Figure 1B**); vB_KpnS_SCNJ1-C had an icosahedral head of 68.9 ± 1.9 nm and long contractile tail of 143.0 ± 1.0 nm (**Figure 1C**); vB_KpnM_SCNJ1-Y revealed an icosahedral head with a diameter of 77.5 ± 6.1 nm and connected tail of 89.0 ± 8.8 nm in length (**Figure 1D**). Under the TEM, vB_KpnA_SCNJ1-Z, vB_KpnS_SCNJ1-C, and vB_KpnM_SCNJ1-Y exhibited *podovirus*/*Autographiviridae*, *siphovirus*, and *myovirus* morphologies, respectively.

### 3.2 Bioinformatics analyses of phage genomes

The complete genome sequences of the three phages were obtained through whole genome sequencing (WGS). vB_KpnA_SCNJ1-Z (Accession no. OQ689084), vB_KpnS_SCNJ1-C (Accession no. OQ718882), and vB_KpnM_SCNJ1-Y (Accession no. OQ689083), possess double-stranded DNA genomes with sizes of 43,428 bp, 46,039 bp, and 50,360 bp, and GC contents of the phages are 54.04%, 47.63%, and 48.52%, respectively.

**Figure 2:**
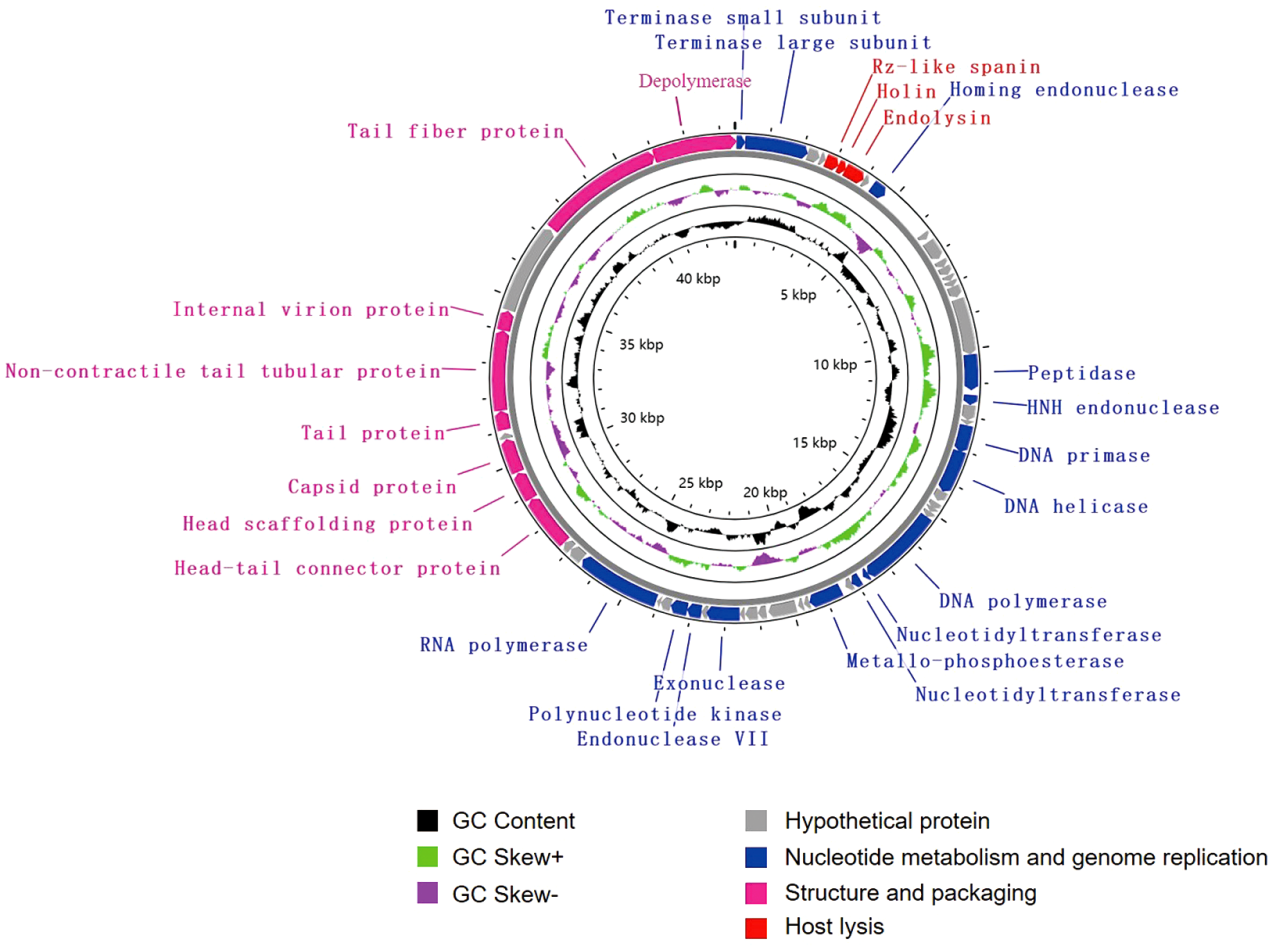
Genome map of vB_KpnA_SCNJ1-Z. Circles are from inside to outside: GC skew plot, G + C% content, tRNA (non-essential), and the ORFs transcribed in clockwise or counterclockwise directions are depicted by different colored arrows based on their function.

The genome of vB_KpnA_SCNJ1-Z contains 57 putative open reading frames (ORFs), with 26 annotated and 31 uncharacterized ORFs (**Figure 2**; **Supplementary Table S2**). The annotated ORFs are grouped into three major functional modules: (i) structure and packaging (8 ORFs), mainly including head and tail-related proteins (ORF48, 49, 52, 53, 56), major capsid (ORF50), and depolymerase (ORF57); (ii) host lysis (3 ORFs), encompassing Rz-like spanin (ORF5), holin (ORF6), and endolysin (ORF7); and (iii) nucleotide metabolism and genome replication (15 ORFs), mainly including terminase (ORF1, 2), endonuclease (ORF9, 19, 41), DNA primase (ORF22), DNA helicase (ORF23), DNA polymerase (ORF28), nucleotidyltransferase (ORF29, 30), and RNA polymerase (ORF45). The genome sequence alignment showed that vB_KpnA_SCNJ1-Z had the highest coverage (91%) and identity (93.07%) with *Klebsiella* virus KpV2883 (Accession no. MT682065) (**Supplementary Figure 1A**), followed by *Klebsiella* phages vB_KpnP_SU552A (Accession no. NC_028870), KP-Rio/2015 (Accession no. NC_047779), and VLC6 (Accession no. MT197176), with identity ranging from 92.46% to 92.8% (coverage 85%) (**Supplementary Figure 2**).

**Figure 3:**
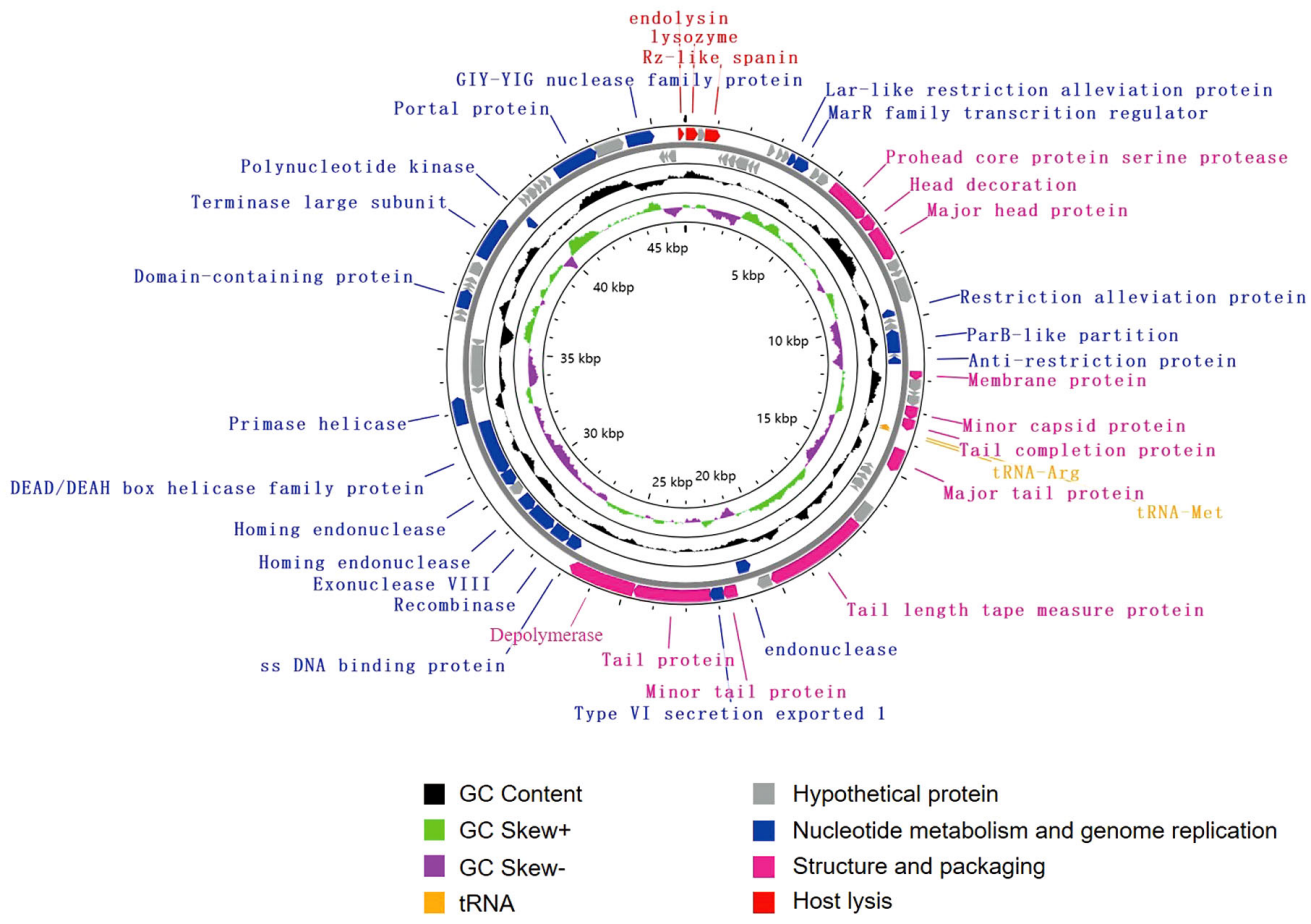
Genome map of vB_KpnS_SCNJ1-C. Circles are from inside to outside: GC skew plot, G + C% content, tRNA (non-essential), and the ORFs transcribed in clockwise or counterclockwise directions are depicted by different colored arrows based on their function.

The genome of vB_KpnS_SCNJ1-C contains 83 putative open reading frames (ORFs), of which 35 are annotated and 48 are uncharacterized (**Figure 3**; **Supplementary Table S3**). These annotated ORFs can be classified into four functional modules: (i) structure and packaging (11 ORFs), mainly comprising head and tail-related proteins (ORF17, 18, 34, 37,44, 47, 49), capsid protein (ORF19, 33), restriction alleviation protein (ORF13, 23), membrane protein (ORF29), and depolymerase (ORF50); (ii) host lysis (3 ORFs), which includes lysozyme (ORF1), Rz-like spanin (ORF3), and endolysin (ORF83); (iii) nucleotide metabolism and genome replication (19 ORFs), mainly including terminase (ORF69), endonuclease (ORF54, 56), single-stranded DNA-binding protein (ORF51), exonuclease (ORF53), and recombinase (ORF52); (iv) tRNA (2 ORFs): tRNA-Met (ORF35) and tRNA-Arg (ORF36). Genome sequence alignment showed that vB_KpnS_SCNJ1-C shared the highest coverage (70%) and identity (94.03%) with *Klebsiella* phage vB_KpnS_MK54 (Accession no. NC_071146) (**Supplementary Figure 1B**), followed by *Vibrio* phage pYD38-A (Accession no. NC_021534), *Aeromonas* phage pIS4-A (Accession no. NC_042037), and *Klebsiella* phage VLCpiS13d (Accession no. NC_071142), with identities ranging from 91.6% to 94.34% (65% coverage) (**Supplementary Figure 3**).

**Figure 4:**
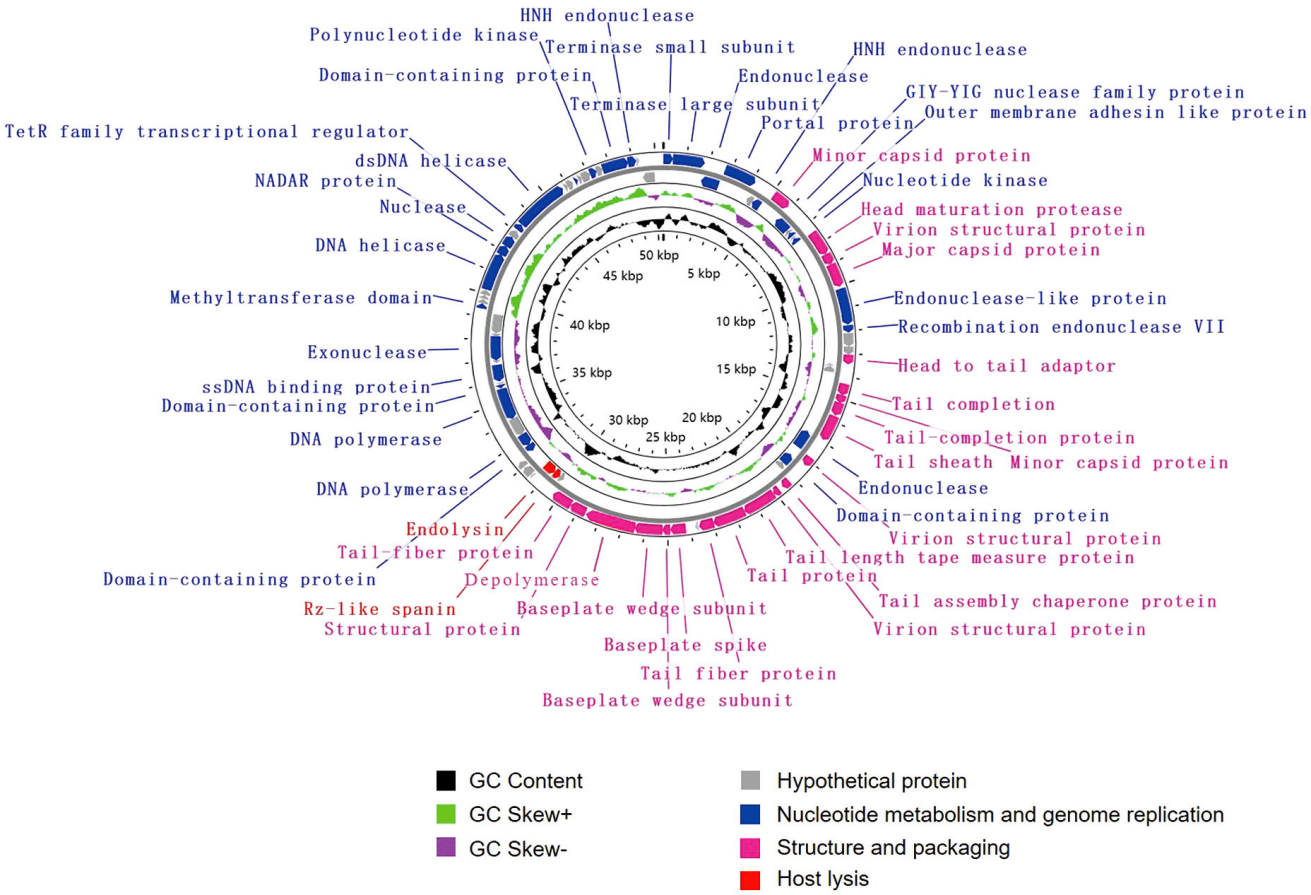
Genome map of vB_KpnM_SCNJ1-Y. Circles are from inside to outside: GC skew plot, G + C% content, tRNA (non-essential), and the ORFs transcribed in clockwise or counterclockwise directions are depicted by different colored arrows based on their function.

The genome of vB_KpnM_SCNJ1-Y contains 80 putative open reading frames (ORFs), with 52 annotated and 28 unannotated ORFs (**Figure 4**; **Supplementary Table S4**). The annotated ORFs are organized into three functional modules: (i) structure and packaging, consisting of 21 ORFs mainly including head and tail-related proteins (ORF13, 20, 25, 26, 31, 33-35, 42), capsid protein (ORF7, 15, 24), baseplate protein (ORF37-39), and depolymerase (ORF40); (ii) host lysis, consisting of 2 ORFs: Rz-like spanin (ORF44) and endolysin (ORF45); and (iii) nucleotide metabolism and genome replication, consisting of 29 ORFs mainly including terminase (ORF1, 2), endonuclease (ORF3, 6, 16, 17, 27, 78), transcriptional regulator (ORF68), DNA helicase (ORF64, 69), DNA polymerase (ORF50, 52), exonuclease (ORF57), and polynucleotide kinase (ORF75). The genome sequence alignment showed that vB_KpnM_SCNJ1-Y shared the highest coverage (69%) and identity (91.24%) with *Klebsiella* phage vB_KpnM_JustaPhage (Accession no. NC_071134.1) (**Supplementary Figure 1C**), followed by *Klebsiella* phage BUCT_49532 (Accession no.NC_071129.1), *Klebsiella* phage 1611E-K2-1 (Accession no.NC_071138.1), and *Escherichia* phage ZCEC13 (GenBank: NC_071140.1), which shared 94.04%, 94.16%, and 92.82% identity with coverage of 71%, 69%, and 69%, respectively (**Supplementary Figure 4**).

**Figure 5:**
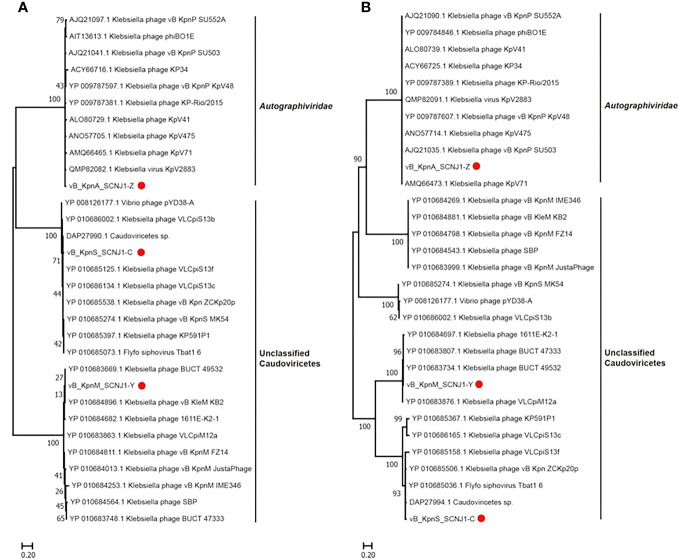
Phylogenetic relationship of vB_KpnA_SCNJ1-Z, vB_KpnS_SCNJ1-C, vB_KpnM_SCNJ1-Y, and other phages within the class *Caudoviricetes*. **(A)** The phylogenetic tree of the major capsid. **(B)** The phylogenetic tree of large terminase. The three phages of the study were marked with red circles. Phylogenetic trees were constructed using MEGA 11.0 by the neighbor-joining method (1000 bootstrap replications). Bootstrap values are shown at the branches. Scale bars below indicate the amino acid substitutions per site.

Phylogenetic trees were constructed using the amino acid sequences of the major capsid and large terminase of representative virus species of the family *Autographiviridae* and unclassified *Caudoviricetes*. In both phylogenetic trees, vB_KpnA_SCNJ1-Z was clustered with viruses within the family *Autographiviridae*, while both vB_KpnS_SCNJ1-C and vB_KpnM_SCNJ1-Y were clustered with unclassified Caudoviricetes within the class *Caudoviricete* (**Figure 5**). According to the genome alignment result, vB_KpnA_SCNJ1-Z shared the highest similarity (91% coverage and 93.07% identity) with *Klebsiella* virus KpV2883, vB_KpnS_SCNJ1-C shared the highest similarity (70% coverage and 94.03% identity) with *Klebsiella* phage vB_KpnS_MK54, and vB_KpnM_SCNJ1-Y shared the highest similarity (69% coverage and 91.24% identity) with *Klebsiella* phage vB_KpnM_JustaPhage. The main species demarcation criterion for bacterial and archaeal viruses is set at overall DNA sequence homology of 95% according to the International Committee on Taxonomy of Viruses (ICTV) (Adriaenssens et al., 2020). Thus, all three phages are designated novel species within the class *Caudoviricetes*. No virulence factors, lysogenic, integrase, or AMRs were detected in the genomes of the three phages, suggesting their potential as virulent phages for use in biological safety applications.

### 3.3 Physiological characterization of phages

**Figure 6:**
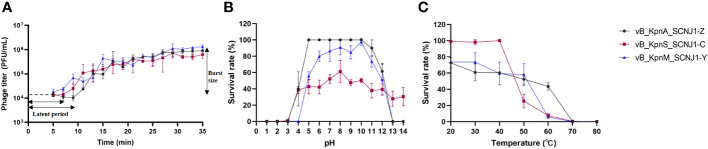
Physiological characterization of phages. **(A)** One-step growth curves of vB_KpnA_SCNJ1-Z, vB_KpnS_SCNJ1-C, and vB_KpnM_SCNJ1-Y. **(B)** pH stabilities of vB_KpnA_SCNJ1-Z, vB_KpnS_SCNJ1-C, and vB_KpnM_SCNJ1-Y. **(C)** Temperature stabilities of vB_KpnA_SCNJ1-Z, vB_KpnS_SCNJ1-C, and vB_KpnM_SCNJ1-Y.

The optimal MOI of vB_KpnA_SCNJ1-Z, vB_KpnS_SCNJ1-C, and vB_KpnM_SCNJ1-Y was determined to be 0.0001, with the highest titers observed at 3.6×10^9^ PFU/mL, 4.3×10^10^ PFU/mL, and 8.2×10^9^ PFU/mL, respectively (**Supplementary Table S5**). Analysis of their one-step growth curves revealed that vB_KpnS_SCNJ1-C and vB_KpnM_SCNJ1-Y had a shorter latent period of 7 min, while the latent period of vB_KpnA_SCNJ1-Z was 9 min. The burst size of vB_KpnA_SCNJ1-Z, vB_KpnS_SCNJ1-C, and vB_KpnM_SCNJ1-Y was determined to be 9.06 ± 0.81 PFU/cell, 6.25 ± 1.58 PFU/cell, and 13.26 ± 2.53 PFU/cell, respectively (**Figure 6A**).

For pH stability testing, both vB_KpnA_SCNJ1-Z and vB_KpnM_SCNJ1-Y exhibited a survival rate of 50%-100% within a wide pH range (pH 5-10), while they were inactivated at pH levels below 3 and above 13. vB_KpnS_SCNJ1-C exhibited a survival rate of 40%-60% within the pH range of 4-14 (**Figure 6B**). For heat resistance testing, approximately 50% of phage vB_KpnA_SCNJ1-Z remained viable at 60°C, while vB_KpnS_SCNJ1-C and vB_KpnM_SCNJ1-Y exhibited survival rates of only 5%-10%. At a higher temperature of 70°C, no viable phages were detected for any of the three phages (**Figure 6C**). Furthermore, incubation with 10% chloroform for 30 min resulted in survival rates of 87%, 78%, and 100% for vB_KpnA_SCNJ1-Z, vB_KpnS_SCNJ1-C, and vB_KpnM_SCNJ1-Y, respectively.

### 3.4 Host range of phages

The host range of the phages was assessed against a panel of 50 clinical bacterial strains, as presented in **Supplementary Table S1**. We found that the three phages did not lyse any other strains except the *K. pneumoniae* NJ09 which is a clone of the host bacterium *K. pneumoniae* SCNJ1.

### 3.5 Antibiofilm activity of phages

**Figure 7:**
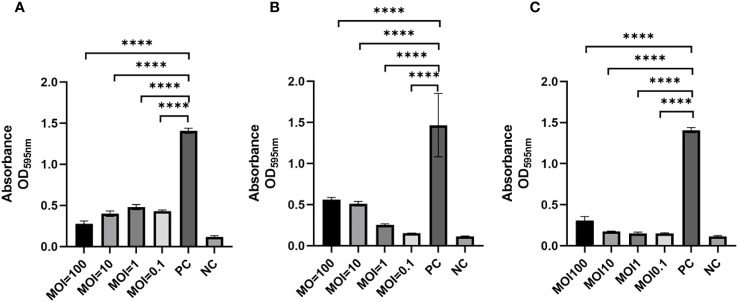
Effects of phages on biofilm formation at different MOIs. Statistically significant differences were determined by a Student’s test using GraphPad Prism v.9.4.0. ****, P<0.0001. **(A)** Effects of vB_KpnA_SCNJ1-Z on biofilm formation. **(B)** Effects of vB_KpnS_SCNJ1-C on biofilm formation. **(C)** Effects of vB_KpnM_SCNJ1-Y on biofilm formation. PC represents the positive control group, and NC indicates the negative control group.

To assess the ability of phages to impede biofilm formation by *K. pneumoniae* SCNJ1, different MOIs (100 - 0.1) of the mixture were incubated for 24 h using 96-well plates. The results showed that biofilm formation was considerably reduced at different MOIs in the phage-treated wells compared to the control group. Specifically, the absorbance value of the vB_KpnA_SCNJ1-Z-treated group (MOI = 100) (**Figure 7A**) was substantially reduced by 80% in comparison to the control group. Similarly, the absorbance value of the vB_KpnS_SCNJ1-C-treated group and vB_KpnM_SCNJ1-Y-treated group (MOI = 0.1) were significantly reduced by 90% and 89% (**Figures 7B, C**), respectively, in comparison to the control group. The results suggested that all three phages have the potential to inhibit the biofilm formation of SCNJ1.

### 3.6 Antibacterial activity of the phages *in vitro*

**Figure 8:**
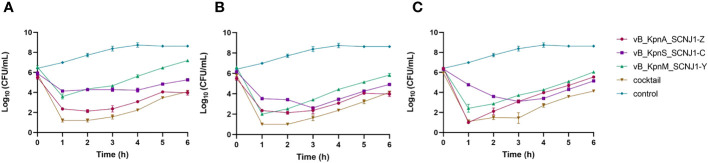
Growth curves of *K*. *pneumoniae* SCNJ1 infected with phages at different MOIs. **(A)** Cell counts of cultures treated with phages with MOI=100. **(B)** Cell counts of cultures treated with phages with MOI=10. **(C)** Cell counts of cultures treated with phages with MOI=1. The control group is added with LB broth instead of phages.

To evaluate the efficiency of phage infection, *K. pneumoniae* SCNJ1 was infected with either a single phage or a phage cocktail consisting of vB_KpnA_SCNJ1-Z, vB_KpnS_SCNJ1-C, and vB_KpnM_SCNJ1-Y in equal proportions, with MOIs of 1, 10, and 100. The inhibitory effects of bacterial growth were assessed by comparing bacterial counts in the presence or absence of phage. All the single phage-treated groups showed a sharp decline in bacterial counts in the first hour at different MOIs, followed by regrowth of the resistant population during the remaining 5 hours of incubation, resulting in bacterial counts of 0.8×10^4^-1.5×10^7^ CFU/mL. However, after 6 hours of incubation, the bacterial counts of all single phage-treated groups were much lower than that of the control group which had bacterial counts of ~ 4×10^8^ CFU/mL. The cocktail-treated group showed a similar distribution of growth inhibition compared to that of every single phage (**Figure 8**).

### 3.7 Phage treatment against SCNJ1 infection in the mouse model

**Figure 9:**
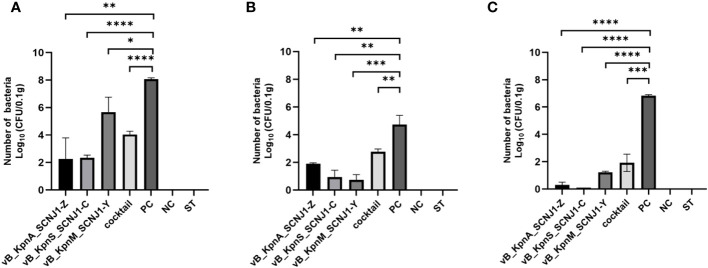
Bacterial loads in the organs of mice infected with *K. pneumoniae* SCNJ1 after treatment with phages for 30 h. **(A)** Bacterial loads in the lungs. **(B)** Bacterial loads in the livers. **(C)** Bacterial loads in the spleens. PC represents the positive control group, NC indicates the negative control group, and ST represents the safe test group. Student’s test was used to determine differences between control and treatment groups using GraphPad Prism v.9.4.0. *, P ≤ 0.05, **, P ≤ 0.01, ***, P ≤ 0.001, ****, P<0.0001.

**Figure 10:**
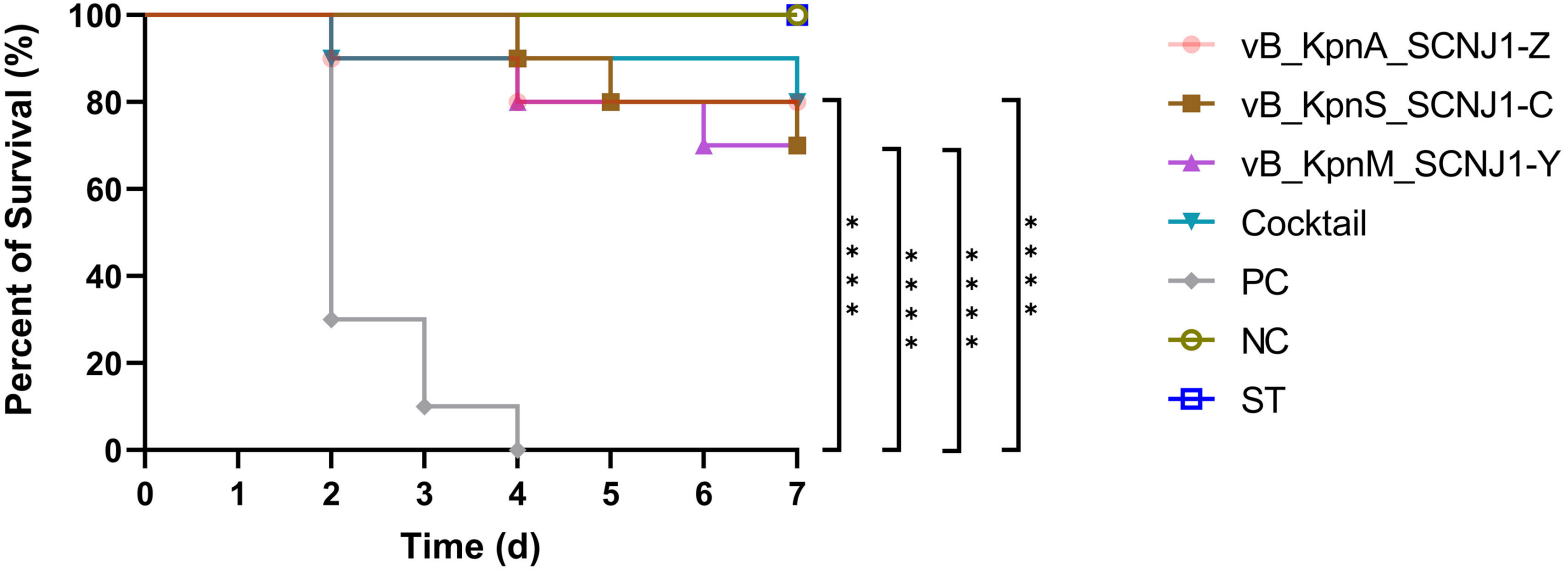
Survival rates of *K. pneumoniae* SCNJ1-infected mice in each group. Each group contained ten mice. Statistical analysis was performed using the Kaplan-Meier method [P < 0.0001, log-rank test]. PC represents the positive control group, NC indicates the negative control group, ST represents the safe test group, ****, P<0.0001.

**Figure 11:**
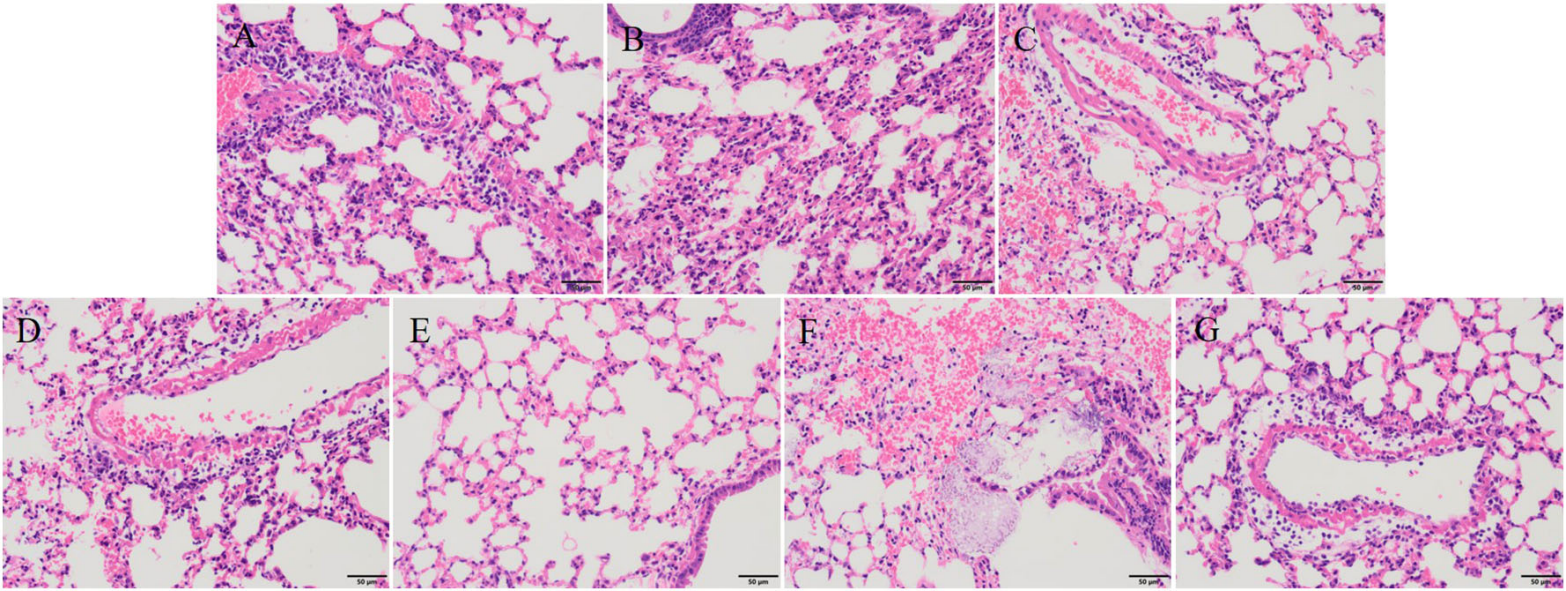
Histopathological analysis of mouse lung tissue (magnification, ×200). **(A)** vB_KpnA_SCNJ1-Z-treated group. **(B)** vB_KpnS_SCNJ1-C-treated group. **(C)** vB_KpnM_SCNJ1-Y-treated group. **(D)** Phage cocktail-treated group. **(E)** The negative control group. **(F)** The positive control group. **(G)** Safe test group.

After 30 hours post-infection, the bacterial counts in the phage-treated groups showed a significant reduction in organs compared to those in the positive control group. For lung, the bacterial loads were approximately 10^2^ CFU/0.1g, 10^2^ CFU/0.1g, 10^6^ CFU/0.1g, and 10^4^ CFU/0.1g in the vB_KpnA_SCNJ1-Z, vB_KpnS_SCNJ1-C, vB_KpnM_SCNJ1-Y, and cocktail groups, respectively, whereas the bacterial load in the positive control group was approximately 10^8^ CFU/0.1g. For the liver, bacterial count was 10^5^ CFU/0.1g in the positive control group while those in the vB_KpnA_SCNJ1-Z, vB_KpnS_SCNJ1-C, vB_KpnM_SCNJ1-Y, and cocktail-treated groups was ~10^2^ CFU/0.1g, ~10 CFU/0.1g, ~10 CFU/0.1g, ~10^3^ CFU/0.1g, respectively. Furthermore, compared to the bacterial load of the positive control group (10^7^ CFU/0.1g), all the phage-treated groups exhibited a substantial reduction, with the bacterial count reaching ≤ ~10^2^ CFU/0.1g in the spleen (**Figure 9**). vB_KpnA_SCNJ1-Z, vB_KpnS_SCNJ1-C, vB_KpnM_SCNJ1-Y, and cocktail-treated groups demonstrated a survival rate of 80%, 70%, 70%, and 80% within 7 days, respectively (**Figure 10**). All mice in the positive control group succumbed to infection, whereas mice in the negative group and the safe test group survived. The histological examination of lung tissues of mice treated with vB_KpnA_SCNJ1-Z, vB_KpnS_SCNJ1-C, vB_KpnM_SCNJ1-Y, and cocktail showed significantly alleviated lesions with less inflammatory cell infiltration around the blood vessels (**Figures 11A–E**), in contrast, the positive control group showed thickened alveolar septum with infiltration of inflammation cells (**Figure 11F**). The lung tissue in the safe test group showed a small number of inflammatory cells in the alveolar (**Figure 11G**).

## 4 Discussion

In recent years, *K. pneumoniae* has emerged as a significant nosocomial and opportunistic pathogen. Antibiotic resistance, with the production of extended-spectrum β-lactamases (ESBL), carbapenemases or aminoglycosides resistance, is common in *K. pneumoniae* (Raouf et al., 2022; Jwair et al., 2023; Khoshnood et al., 2023), which brings big challenges to the therapy of infection of *K. pneumoniae*. The emergence of carbapenem-resistant hypervirulent *K. pneumoniae* (CR-hvKP) has exhibited prevalence since 2010 (Lan et al., 2021). The rapid spread and outbreaks of CR-hvKP in nosocomial infection present a formidable hurdle for effective infection control. The high level of resistance and pathogenicity often results in poor prognosis for CR-hvKP infection (Gu et al., 2018; Zhu et al., 2022). The occurrence of colistin- or tigecycline-resistant CR-hvKP clinical isolates calls for the development of novel therapeutic strategies against CR-hvKP infections (Li et al., 2019; Zhang et al., 2021b; Zhang et al., 2021c). Phages are targeted viruses with a variety of antimicrobial effects that specifically lyse host bacteria and have become a promising replacement for bacterial therapy after antibiotics (Cano et al., 2021; Li et al., 2022; Lu et al., 2022).

In this study, we isolated three novel lytic phages from sewage and river water, against a strain of ST29 K54 CR-hvKP, *K. pneumonia* SCNJ1. The three phages formed clear plaques on the lawns of host cells with a transparent halo around the plaque. A head-tail morphology was observed through a transmission electronic microscope for all three phages, which indicated they all belong to the class *Caudoviricetes.* This classification aligns with other phages known to infect *K. pneumoniae* (Tan et al., 2019; Anand et al., 2020; Fang and Zong, 2022). Specifically, vB_KpnA_SCNJ1-Z, vB_KpnS_SCNJ1-C, and vB_KpnM_SCNJ1-Y exhibited *podovirus*/*Autographiviridae*, *siphovirus*, and *myovirus* morphologies, respectively. The three phages exhibited a restricted host range, as they were only able to infect and lyse one out of the 50 tested clinical bacterial strains, this result is similar to that of another lytic *K. pneumoniae* phage vB_KpnP_Bp5, in which 36 test strains could not be lysed by vB_KpnP_Bp5 except the host bacteria (Zhang et al., 2021a). This narrow host range may potentially pose limitations to their future applications for phages with a broader host spectrum are generally more desirable. The optimal MOI of the three phages is 0.0001, with the highest phage titers. Compared to previously reported *K. pneumoniae* phages which exhibited optimal MOIs range from 10-0.001, the phages of our study displayed a lower MOI (0.0001). A lower MOI means fewer phages are required to lyse the same number of bacteria, making it the preferred choice for reducing phage production and application costs (Tan et al., 2019; Anand et al., 2020; Zhang et al., 2021a; Pu et al., 2022). For the one-step growth curves, the latent period of vB_KpnA_SCNJ1-Z, vB_KpnS_SCNJ1-C, and vB_KpnM_SCNJ1-Y were 9 min, 7 min, and 7 min, respectively. The short latent period of a phage reflects its rapid adsorption to the host surface. Short-latency phages have been shown to lyse more bacterial cells in a certain time, indicating their potential suitability for biocontrol application (Abedon, 1989). The thermal and pH stabilities of the three phages were found to be similar to that of other *Klebsiella* phages with their tolerance to a wide pH range (3-14) and excellent thermal stabilities (20-60°C) (Anand et al., 2020; Pu et al., 2022). These characteristics suggested that the three phages possess the potential to serve as stable biological agents. The formation of biofilm by *K. pneumoniae* strain SCNJ1 was significantly decreased at different MOIs of the three phages. This reduction may be attributed to the enzymatic activity of depolymerases encoded by the three phages of our study, which facilitate the degradation of the extracellular matrix of the biofilm (Gordillo Altamirano and Barr, 2019). All three phages are insensitive to chloroform, which indicated the three phages lack lipids.

Phylogenetic analysis demonstrated that vB_KpnA_SCNJ1-Z belongs to the family *Autographiviridae* within class *Caudoviricetes*, while vB_KpnS_SCNJ1-C and vB_KpnM_SCNJ1-Y are unclassified *Caudoviricetes*. Genomic analysis showed vB_KpnA_SCNJ1-Z had the highest coverage (91%) and identity (93.07%) with *Klebsiella* virus KpV2883, vB_KpnS_SCNJ1-C shared the highest coverage (70%) and identity (94.03%) with *Klebsiella* phage vB_KpnS_MK54, vB_KpnM_SCNJ1-Y shared the highest coverage (69%) and identity (91.24%) with *Klebsiella* phage vB_KpnM_JustaPhage. The main species demarcation criterion for bacterial and archaeal viruses is set at overall DNA sequence homology of 95% according to the ICTV (Adriaenssens et al., 2020). Therefore, the three phages can be described as representatives of new species within the class *Caudoviricetes*. No virulence factors, lysogenic, integrase, or AMRs were detected in the three phages, which indicates their potential for use in phage therapy. Similar to phage BUCT54, which is a lytic phage against multi-drug-resistant *K. pneumoniae* (MDR-KP), two tRNA, i.e. tRNA-Met (ORF35) and tRNA-Arg (ORF36) were found in the genome of vB_KpnS_SCNJ1-C, which indicated the replication of vB_KpnS_SCNJ1-C may need a large amount of methionine (Met) and arginine (Arg) and the two tRNA may help to transfer the two amino acids in the host cell (Pu et al., 2022).

We have assessed the effect of the three phages against *K. pneumoniae* SCNJ1 *in vitro* and *in vivo*. *In vitro*, the number of *K. pneumoniae* strain SCNJ1 in all phage-treated groups declined in the first hour and regrew in the following hours, indicating an arms-race phenomenon between bacteria and phages (Hampton et al., 2020). Compared with the control group, the single phage-treated groups and the cocktail-treated group at different MOIs could inhibit the growth rate of *K. pneumoniae* strain SCNJ1, which showed the possibility of the three phages could be used as a biocontrol agent to contain the spread of *K. pneumoniae in vitro*. *In vivo*, we used a mouse model to examine the therapeutic effect of phages on *K. pneumoniae* SCNJ1 and evaluate the potential of the three phages for clinical application. We have observed that the intranasal administration of high-titer of the single phage or cocktail (1×10^9^ PFU/mL) within 30 hours post-infection can effectively reduce the bacterial load in the lung, liver, and spleen. A survival rate of approximately 80% was observed in both the phage cocktail-treated group and the single phage-treated group. Compared to other phages used in the treatment of infection of *K. pneumoniae*, such as phage BUCT54, Pharr, and ϕKpNIH-2, which have demonstrated 100% survival rates in infected mice, our mouse experiments yielded a maximum survival rate of 80% (Hesse et al., 2021; Pu et al., 2022). This disparity may be attributed to inadequate phage dosage or an inappropriate MOI of phage-to-bacteria that was applied in our study. Histopathological analysis revealed that the groups treated with phages exhibited substantially reduced damage compared to the positive control group, aligning with the findings regarding bacterial loads in organs of mice, indicating that phages possess the ability to effectively eradicate *K. pneumoniae in vivo*, similar to the results of several related studies of phage therapy (Hua et al., 2017; Teng et al., 2022). The mice in the phage-treated groups have mild lesions that may be due to endotoxins released from the lysed bacteria (Anand et al., 2020). Although there are a small number of inflammatory cells in the safe test group, it does not affect the healthy life activity of the mice (Pu et al., 2022). For the *in vitro* test, we used one-way analysis of variance to compare significant differences within bacterial loads of the treatment groups. There was almost no statistical significance between the single phage-treated groups and the cocktail-treated group (**Supplementary Table S6**–**8**; **Figure 8**); for the *in vivo* test, survival curve analyses were performed using Kaplan–Meier survival analysis (**Figure 10**). The differences in survival rates of 80% and 70% were not statistically significant; for bacterial load in various organs, the cocktail-treated group did not exhibit the lowest bacterial load among all treatment groups. In summary, compared to single phage treatment, cocktail therapy lacked significant advantages, possibly due to the high specificity of the three phages sharing the same receptor, leading to an absence of synergistic effects. Nevertheless, both single phage and cocktail treatments demonstrated robust *in vitro* and *in vivo* antibacterial efficacy, suggesting the three phages can potentially be used as an alternative therapy for CR-hvKP infection.

## 5 Conclusions

In this study, we have successfully isolated and characterized three novel lytic phages that specifically target a strain of ST29 K54 CR-hvKP *K. pneumonia* SCNJ1. Our findings revealed that the phages exhibited excellent tolerance to a broad pH range and thermal stabilities, biological safety, and satisfactory therapeutic efficacy in BALB/c mice. Besides, all three phages were effective in reducing biofilm formation by *K. pneumoniae* strain SCNJ1, and no genes for virulence, lysogenic, integrase, or AMRs were found in their genomes indicating their great potential as a promising alternative for antimicrobial therapy.

## Data availability statement

The sequences of vB_KpnA_SCNJ1-Z, vB_KpnM_SCNJ1-Y, and vB_KpnS_SCNJ1-C have been deposited in the GenBank under accession numbers OQ689084, OQ689083, and OQ718882.

## Ethics statement

The animal study was approved by The Ethics Committee of Experimental Animals, Southwest Medical University (swmu20230036). The study was conducted in accordance with the local legislation and institutional requirements.

## Author contributions

LZ: Conceptualization, Supervision, Writing – review & editing. CF: Formal Analysis, Methodology, Writing – original draft. XD: Formal Analysis, Resources, Writing – review & editing. LX: Methodology, Software, Writing – review & editing. YQ: Formal Analysis, Resources, Writing – review & editing. MY: Formal Analysis, Resources, Writing – review & editing. YF: Formal Analysis, Resources, Writing – review & editing. YL: Writing – review & editing.

## References

[B1] Abedon S. T. (1989). Selection for bacteriophage latent period length by bacterial density: A theoretical examination. Microb. Ecol. 18, 79–88. doi: 10.1007/BF02030117 24196124

[B2] Adams M. H. Park B. H. (1956). An enzyme produced by a phage-host cell system. II. The properties of the polysaccharide depolymerase. Virology 2, 719–736. doi: 10.1016/0042-6822(56)90054-X 13392519

[B3] Adams M. H. J. B. (1959). Bacteriophages. doi: 10.5962/bhl.title.6966

[B4] Adriaenssens E. M. Sullivan M. B. Knezevic P. Van Zyl L. J. Sarkar B. L. Dutilh B. E.. (2020). Taxonomy of prokaryotic viruses: 2018-2019 update from the ICTV Bacterial and Archaeal Viruses Subcommittee. Arch. Virol. 165, 1253–1260. doi: 10.1007/s00705-020-04577-8 32162068 PMC13502909

[B5] Anand T. Virmani N. Kumar S. Mohanty A. K. Pavulraj S. Bera B. C.. (2020). Phage therapy for treatment of virulent *Klebsiella pneumoniae* infection in a mouse model. J. Glob Antimicrob. Resist. 21, 34–41. doi: 10.1016/j.jgar.2019.09.018 31604128

[B6] Cano E. J. Caflisch K. M. Bollyky P. L. Van Belleghem J. D. Patel R. Fackler J.. (2021). Phage therapy for limb-threatening prosthetic knee *klebsiella pneumoniae* infection: case report and *in vitro* characterization of anti-biofilm activity. Clin. Infect. Dis. 73, e144–e151. doi: 10.1093/cid/ciaa705 32699879 PMC8246933

[B7] Chadha P. Katare O. P. Chhibber S. (2017). Liposome loaded phage cocktail: Enhanced therapeutic potential in resolving *Klebsiella pneumoniae* mediated burn wound infections. Burns 43, 1532–1543. doi: 10.1016/j.burns.2017.03.029 28502784

[B8] Chen Y. Li W. Shi K. Fang Z. Yang Y. Zhang R. (2023). Isolation and characterization of a novel phage belonging to a new genus against *Vibrio* parahaemolyticus. Virol. J. 20, 81. doi: 10.1186/s12985-023-02036-9 37127579 PMC10152775

[B9] Fang Q. Zong Z. (2022). Lytic Phages against ST11 K47 Carbapenem-Resistant *Klebsiella pneumoniae* and the Corresponding Phage Resistance Mechanisms. mSphere 7, e0008022. doi: 10.1128/msphere.00080-22 35255715 PMC9044933

[B10] Fu J. Li Y. Zhao L. Wu C. He Z. (2023). Characterization of vB_ValM_PVA8, a broad-host-range bacteriophage infecting *Vibrio alginolyticus* and *Vibrio parahaemolyticus* . Front. Microbiol. 14, 1105924. doi: 10.3389/fmicb.2023.1105924 37250064 PMC10213691

[B11] Gordillo Altamirano F. L. Barr J. J. (2019). Phage therapy in the postantibiotic era. Clin. Microbiol. Rev. 32, e00066-18. doi: 10.1128/CMR.00066-18 30651225 PMC6431132

[B12] Gu D. Dong N. Zheng Z. Lin D. Huang M. Wang L.. (2018). A fatal outbreak of ST11 carbapenem-resistant hypervirulent *Klebsiella pneumoniae* in a Chinese hospital: a molecular epidemiological study. Lancet Infect. Dis. 18, 37–46. doi: 10.1016/S1473-3099(17)30489-9 28864030

[B13] Hampton H. G. Watson B. N. J. Fineran P. C. (2020). The arms race between bacteria and their phage foes. Nature 577, 327–336. doi: 10.1038/s41586-019-1894-8 31942051

[B14] Hesse S. Malachowa N. Porter A. R. Freedman B. Kobayashi S. D. Gardner D. J.. (2021). Bacteriophage treatment rescues mice infected with multidrug-resistant *Klebsiella pneumoniae* ST258. mBio 12, e00034-21. doi: 10.1128/mBio.00034-21 33622728 PMC8545083

[B15] Hua Y. Luo T. Yang Y. Dong D. Wang R. Wang Y.. (2017). Phage therapy as a promising new treatment for lung infection caused by carbapenem-resistant *Acinetobacter baumannii* in mice. Front. Microbiol. 8, 2659. doi: 10.3389/fmicb.2017.02659 29375524 PMC5767256

[B16] Hyman P. Abedon S. T. (2009). Practical methods for determining phage growth parameters. Methods Mol. Biol. 501, 175–202. doi: 10.1007/978-1-60327-164-6_18 19066822

[B17] Jwair N. A. Al-Ouqaili M. T. S. Al-Marzooq F. (2023). Inverse association between the existence of CRISPR/cas systems with antibiotic resistance, extended spectrum β-lactamase and carbapenemase production in multidrug, extensive drug and pandrug-resistant *Klebsiella pneumoniae* . Antibiotics (Basel) 12, 980. doi: 10.3390/antibiotics12060980 PMC10295211 37370299

[B18] Khoshnood S. Akrami S. Saki M. Motahar M. Masihzadeh S. Daneshfar S.. (2023). Molecular evaluation of aminoglycosides resistance and biofilm formation in *Klebsiella pneumoniae* clinical isolates: A cross-sectional study. Health Sci. Rep. 6, e1266. doi: 10.1002/hsr2.1266 37205937 PMC10190123

[B19] Lan P. Jiang Y. Zhou J. Yu Y. (2021). A global perspective on the convergence of hypervirulence and carbapenem resistance in *Klebsiella pneumoniae* . J. Glob Antimicrob. Resist. 25, 26–34. doi: 10.1016/j.jgar.2021.02.020 33667703

[B20] Li J. Huang Z. Y. Yu T. Tao X. Y. Hu Y. M. Wang H. C.. (2019). Isolation and characterization of a sequence type 25 carbapenem-resistant hypervirulent *Klebsiella pneumoniae* from the mid-south region of China. BMC Microbiol. 19, 219. doi: 10.1186/s12866-019-1593-5 31533609 PMC6749629

[B21] Li Y. Lv P. Shi D. Zhao H. Yuan X. Jin X.. (2022). A cocktail of three virulent phages controls multidrug-resistant *Salmonella enteritidis* infection in poultry. Front. Microbiol. 13, 940525. doi: 10.3389/fmicb.2022.940525 35875532 PMC9298555

[B22] Li N. Zeng Y. Bao R. Zhu T. Tan D. Hu B. (2021). Isolation and characterization of novel phages targeting pathogenic *Klebsiella pneumoniae* . Front. Cell Infect. Microbiol. 11, 792305. doi: 10.3389/fcimb.2021.792305 34926329 PMC8677704

[B23] Lin T. L. Hsieh P. F. Huang Y. T. Lee W. C. Tsai Y. T. Su P. A.. (2014). Isolation of a bacteriophage and its depolymerase specific for K1 capsule of *Klebsiella pneumoniae*: implication in typing and treatment. J. Infect. Dis. 210, 1734–1744. doi: 10.1093/infdis/jiu332 25001459

[B24] Lu B. Yao X. Han G. Luo Z. Zhang J. Yong K.. (2022). Isolation of *Klebsiella pneumoniae* Phage vB_KpnS_MK54 and Pathological Assessment of Endolysin in the Treatment of Pneumonia Mice Model. Front. Microbiol. 13, 854908. doi: 10.3389/fmicb.2022.854908 35387089 PMC8978833

[B25] Mulani M. S. Kumkar S. N. Pardesi K. R. (2022). Characterization of novel *Klebsiella phage* PG14 and its antibiofilm efficacy. Microbiol. Spectr. 10, e0199422. doi: 10.1128/spectrum.01994-22 36374021 PMC9769620

[B26] Pu M. Li Y. Han P. Lin W. Geng R. Qu F.. (2022). Genomic characterization of a new phage BUCT541 against *Klebsiella pneumoniae* K1-ST23 and efficacy assessment in mouse and Galleria mellonella larvae. Front. Microbiol. 13, 950737. doi: 10.3389/fmicb.2022.950737 36187954 PMC9523250

[B27] Raouf F. E. A. Benyagoub E. Alkhudhairy M. K. Akrami S. Saki M. (2022). Extended-spectrum beta-lactamases among *Klebsiella pneumoniae* from Iraqi patients with community-acquired pneumonia. Rev. Assoc. Med. Bras. (1992) 68, 833–837. doi: 10.1590/1806-9282.20220222 35766700 PMC9575887

[B28] Russo T. A. Marr C. M. (2019). Hypervirulent *Klebsiella pneumoniae* . Clin. Microbiol. Rev. 32, e00001-19. doi: 10.1128/CMR.00001-19 31092506 PMC6589860

[B29] Serban D. Popa Cherecheanu A. Dascalu A. M. Socea B. Vancea G. Stana D.. (2021). Hypervirulent *Klebsiella pneumoniae* endogenous endophthalmitis-A global emerging disease. Life (Basel) 11, 676. doi: 10.3390/life11070676 PMC8304989 34357049

[B30] Shang Y. Sun Q. Chen H. Wu Q. Chen M. Yang S.. (2021). Isolation and characterization of a novel *Salmonella* phage vB_SalP_TR2. Front. Microbiol. 12, 664810. doi: 10.3389/fmicb.2021.664810 34234757 PMC8256156

[B31] Stone E. Campbell K. Grant I. Mcauliffe O. (2019). Understanding and Exploiting Phage-Host Interactions. Viruses 11, 567. doi: 10.3390/v11060567 PMC6630733 31216787

[B32] Tan D. Zhang Y. Cheng M. Le S. Gu J. Bao J.. (2019). Characterization of *Klebsiella pneumoniae* ST11 Isolates and Their Interactions with Lytic Phages. Viruses 11:1080. doi: 10.3390/v11111080 PMC6893751 31752386

[B33] Tan D. Zhang Y. Qin J. Le S. Gu J. Chen L. K.. (2020). A Frameshift Mutation in wcaJ Associated with Phage Resistance in *Klebsiella pneumoniae* . Microorganisms 8, 378. doi: 10.3390/microorganisms8030378 PMC7142929 32156053

[B34] Teng F. Xiong X. Zhang S. Li G. Wang R. Zhang L.. (2022). Efficacy Assessment of Phage Therapy in Treating *Staphylococcus aureus*-Induced Mastitis in Mice. Viruses 14, 620. doi: 10.3390/v14030620 PMC8954217 35337027

[B35] Torkashvand N. Kamyab H. Shahverdi A. R. Khoshayand M. R. Sepehrizadeh Z. (2023). Isolation, characterization, and genome analysis of a broad host range *Salmonella* phage vB_SenS_TUMS_E4: a candidate bacteriophage for biocontrol. Vet. Res. Commun. 47, 1493–1503. doi: 10.1007/s11259-023-10105-1 37097546

[B36] Yu F. Lv J. Niu S. Du H. Tang Y. W. Bonomo R. A.. (2018). *In Vitro* Activity of Ceftazidime-Avibactam against Carbapenem-Resistant and Hypervirulent *Klebsiella pneumoniae* Isolates. Antimicrob Agents Chemother 62, e01031–18. doi: 10.1128/AAC.01031-18 29891605 PMC6105775

[B37] Yuan Y. Li Y. Wang G. Li C. Chang Y. F. Chen W.. (2019). bla(NDM-5) carried by a hypervirulent *Klebsiella pneumoniae* with sequence type 29. Antimicrob. Resist. Infect. Control 8, 140. doi: 10.1186/s13756-019-0596-1 31452874 PMC6701021

[B38] Zhang Y. Wang X. Wang Q. Chen H. Li H. Wang S.. (2021b). Emergence of tigecycline nonsusceptible and IMP-4 carbapenemase-producing K2-ST65 hypervirulent *Klebsiella pneumoniae* in China. Microbiol. Spectr. 9, e0130521. doi: 10.1128/Spectrum.01305-21 34704778 PMC8549734

[B39] Zhang Y. Wang X. Wang S. Sun S. Li H. Chen H.. (2021c). Emergence of colistin resistance in carbapenem-resistant hypervirulent *Klebsiella pneumoniae* under the pressure of tigecycline. Front. Microbiol. 12, 756580. doi: 10.3389/fmicb.2021.756580 34925264 PMC8672221

[B40] Zhang C. Yuan J. Guo C. Ge C. Wang X. Wei D.. (2021a). Identification and complete genome of lytic “Kp34likevirus” phage vB_KpnP_Bp5 and therapeutic potency in the treatment of lethal *Klebsiella pneumoniae* infections in mice. Virus Res. 297, 198348. doi: 10.1016/j.virusres.2021.198348 33631221

[B41] Zhu J. Jiang X. Zhao L. Li M. (2022). An outbreak of ST859-K19 carbapenem-resistant hypervirulent *Klebsiella pneumoniae* in a chinese teaching hospital. mSystems 7, e0129721. doi: 10.1128/msystems.01297-21 35574716 PMC9239081

[B42] Zhu J. Wang T. Chen L. Du H. (2021). Virulence factors in hypervirulent *Klebsiella pneumoniae* . Front. Microbiol. 12, 642484. doi: 10.3389/fmicb.2021.642484 33897652 PMC8060575

