# Supplementary material for: Isolation and characterization of three novel lytic phages against K54 serotype carbapenem-resistant hypervirulent Klebsiella pneumoniae
Source: Front Cell Infect Microbiol. 2023 Dec 12;13:1265011. doi: 10.3389/fcimb.2023.1265011 (PMC10749971; doi:10.3389/fcimb.2023.1265011)
Supplement: Supplementary file 1 [file DataSheet_1.pdf]

Supplementary Material

Supplemental Table S1. The host range of vB_KpnA_SCNJ1-Z, vB_KpnS_SCNJ1-C, vB_KpnM_SCNJ1-Y.

Genus/Species	Bacterial Strain	Spot assay			Plaque assay		
		phage Z	phage C	phage Y	phage Z	phage C	phage Y
Acinetobacter							
Acinetobacter baumannii	SCLZ32	–	–	–	–	–	–
Enterobacter							
Enterobacter cloacae	NJ07	–	–	–	–	–	–
Kluyvera							
Kluyvera cryocrescens	W13	–	–	–	–	–	–
Citrobacter							
Citrobacter braakii	R53	–	–	–	–	–	–
Raoultella							
Raoultella ornithinolytica	W01	–	–	–	–	–	–
Kerstersia							
Kerstersia gyiorum	KG1	–	–	–	–	–	–
Morganella							
Morganella morganii strain	T14	–	–	–	–	–	–
Providencia							
Providencia alcalifaciens	T18	–	–	–	–	–	–
Escherichia							
Escherichia coli	NJ06	–	–	–	–	–	–
Escherichia coli	R49	–	–	–	–	–	–
Escherichia coli	T10	–	–	–	–	–	–
Escherichia coli	T25	–	–	–	–	–	–
Escherichia coli	T33	–	–	–	–	–	–
Escherichia coli	T34	–	–	–	–	–	–
Providencia sp.	T1	–	–	–	–	–	–
Providencia sp.	T12	–	–	–	–	–	–
Citrobacter sp.	T20	–	–	–	–	–	–
Klebsiella							
Klebsiella pneumoniae	KPN1050	–	–	–	–	–	–
Klebsiella pneumoniae	NJ13	–	–	–	–	–	–
Klebsiella pneumoniae	N4	–	–	–	–	–	–
Klebsiella pneumoniae	R50	–	–	–	–	–	–
Klebsiella pneumoniae	SCPZ	–	–	–	–	–	–
Klebsiella pneumoniae	NJ12	–	–	–	–	–	–
Klebsiella pneumoniae	NC	–	–	–	–	–	–

Klebsiella pneumoniae	NJ10	−	−	−	−	−	−
Klebsiella pneumoniae	NJ09	+	+	+	+	+	+
Klebsiella pneumoniae	NJ08	−	−	−	−	−	−
Klebsiella pneumoniae	N2	−	−	−	−	−	−
Klebsiella pneumoniae	NJ11	−	−	−	−	−	−
Klebsiella pneumoniae	T3	−	−	−	−	−	−
Klebsiella pneumoniae	T4	−	−	−	−	−	−
Klebsiella pneumoniae	T5	−	−	−	−	−	−
Klebsiella pneumoniae	T6	−	−	−	−	−	−
Klebsiella variicola	T9	−	−	−	−	−	−
Klebsiella pneumoniae	T13	−	−	−	−	−	−
Klebsiella pneumoniae	T15	−	−	−	−	−	−
Klebsiella pneumoniae	T16	−	−	−	−	−	−
Klebsiella pneumoniae	T21	−	−	−	−	−	−
Klebsiella pneumoniae	T22	−	−	−	−	−	−
Klebsiella pneumoniae	T23	−	−	−	−	−	−
Klebsiella pneumoniae	T24	−	−	−	−	−	−
Klebsiella pneumoniae	T26	−	−	−	−	−	−
Klebsiella pneumoniae	T27	−	−	−	−	−	−
Klebsiella pneumoniae	T28	−	−	−	−	−	−
Klebsiella pneumoniae	T29	−	−	−	−	−	−
Klebsiella pneumoniae	T30	−	−	−	−	−	−
Klebsiella pneumoniae	T31	−	−	−	−	−	−
Klebsiella pneumoniae	T32	−	−	−	−	−	−
Klebsiella pneumoniae	T35	−	−	−	−	−	−
Klebsiella pneumoniae	T36	−	−	−	−	−	−

phage Z represents vB_KpnA_SCNJ1-Z; phage C represents vB_KpnS_SCNJ1-C; phage Y represents vB_KpnM_SCNJ1-Y.

Supplemental Table S2. Annotated ORFs in the genome of vB_KpnA_SCNJ1-Z

ORF	Strand	Start	Stop	Length (AA)	Putative function	Best-match BLASTp result	Query cover (%)	E-values	Identity (%)	Accession
ORF1	+	46	303	85	Terminase small subunit	*Acinetobacter* phage vB_AbaA_LLY	100	4e-51	100	WEV89139.1
ORF2	+	303	2159	618	Terminase large subunit	*Klebsiella* phage VLCpiA1j	100	0.0	98.87	UVX31342.1
ORF3	+	2159	2533	124	Hypothetical protein	*Klebsiella* phage KpV71	100	4e-81	98.39	YP_009302752.1
ORF4	+	2545	2727	60	Hypothetical protein	*Klebsiella* phage KpV41	100	9e-32	100	YP_009188793.1
ORF5	+	2727	3131	134	Rz-like spanin	*Klebsiella* phage vB_KpnP_KpV48	100	1e-87	100	YP_009787610.1
ORF6	+	3124	3375	83	Holin	*Klebsiella* phage Kp2	100	4e-52	100	YP_009188365.1
ORF7	+	3359	3967	202	Endolysin	*Klebsiella* phage 6995	100	6e-141	96.04	URY99719.1
ORF8	+	3969	4154	61	Hypothetical protein	*Klebsiella* phage KPR2	100	1e-27	93.44	QCG76631.1
ORF9	+	4284	4739	151	Homing endonuclease	*Caudoviricetes* sp.	100	4e-100	92.72	DAV54577.1
ORF10	+	6324	6521	65	Hypothetical protein	*Klebsiella* phage VLCpiA1h	100	5e-37	95.38	UVX30546.1
ORF11	+	6601	7197	198	Hypothetical protein	Bacteriophage sp.	100	3e-132	95.45	UVX36435.1
ORF12	+	7259	7480	73	Hypothetical protein	*Klebsiella* phage F19	100	2e-44	94.52	YP_009006025.1
ORF13	+	7473	7736	87	Hypothetical protein	*Klebsiella* phage FK1979	100	3e-53	95.40	UPW35189.1
ORF14	+	7745	7924	59	Hypothetical protein	*Staphylococcus epidermidis*	100	5e-35	98.31	MCC3681373.1
ORF15	+	7921	8109	62	Hypothetical protein	Not hit	-	-	-	-
ORF16	+	8103	8447	114	Hypothetical protein	*Klebsiella* phage KP34	100	3e-74	94.74	YP_003347659.1
ORF17	+	8494	10164	556	Hypothetical protein	*Klebsiella* phage VLC5	100	0.0	93.00	QIW86379.1
ORF18	+	10164	11207	347	Peptidase	*Klebsiella* phage BUCT86	100	0.0	97.98	UFX84378.1
ORF19	+	11352	11645	97	HNH endonuclease	*Klebsiella* phage CX1	100	4e-65	97.94	QIN95045.1
ORF20	+	11648	12100	150	Hypothetical protein	*Klebsiella* phage SRD2021	98	5e-94	92.57	QWY13503.1
ORF21	+	12093	12239	48	Hypothetical protein	*Klebsiella* phage VLC5	100	8e-21	91.67	QIW86382.1
ORF22	+	12249	13049	266	DNA primase	*Caudoviricetes* sp.	99	0.0	98.49	DAW25729.1
ORF23	+	13030	14310	426	DNA helicase	*Klebsiella* phage pKP-M186-2.2	99	0.0	99.53	UZN24299.1
ORF24	+	14303	14629	108	Hypothetical protein	*Klebsiella* phage VLC5	100	2e-71	97.22	QIW86387.1
ORF25	+	14680	14901	73	Hypothetical protein	*Klebsiella* phage Kp2	100	4e-43	97.26	QWT56607.1
ORF26	+	14859	15014	51	Hypothetical protein	*Klebsiella* phage KPPK108.1	100	1e-27	98.04	UFK09481.1
ORF27	+	15011	15193	60	Hypothetical protein	*Klebsiella* phage vB_KpnP_KpV48	100	3e-35	96.67	YP_009787573.1
ORF28	+	15190	17601	803	DNA polymerase	*Caudoviricetes* sp.	100	0.0	99.38	DAW28284.1
ORF29	+	17598	17762	54	Nucleotidyltransferase	Bacteriophage sp.	57	3e-11	100	UVX34761.1
ORF30	+	17878	18147	89	Nucleotidyltransferas	*Caudoviricetes* sp.	100	4e-56	95.51	DAW27056.1
ORF31	+	18137	18358	73	Hypothetical protein	*Klebsiella* phage VLCpiA1m	100	6e-44	98.63	UVX29183.1
ORF32	+	18520	19500	326	Metallo-phosphoesterase	*Klebsiella* phage KP-Rio/2015	100	0.0	100	YP_009787365.1
ORF33	+	19497	19676	59	Hypothetical protein	*Klebsiella* phage Kp2	100	8e-34	96.61	YP_009188334.1
ORF34	+	19689	19856	55	Hypothetical protein	*Klebsiella* phage vB_KpnP_KpV48	100	3e-27	100	YP_009787578.1
ORF35	+	19908	20741	277	Hypothetical protein	*Klebsiella* phage KpV475	100	0.0	97.83	YP_009280690.1
ORF36	+	20794	21051	85	Hypothetical protein	*Klebsiella* phage CX1	100	1e-48	96.43	QIN95059.1
ORF37	+	21051	21422	123	Hypothetical protein	*Klebsiella* phage BUCT631	100	7e-78	97.56	WAK45666.1
ORF38	+	21425	21583	52	Hypothetical protein	*Klebsiella* phage vB_KpnP_SU552A	100	3e-28	100	YP_009204817.1
ORF39	+	21583	22551	322	Exonuclease	*Caudoviricetes* sp.	100	0.0	99.38	DAO86509.1
ORF40	+	22508	22711	67	Hypothetical protein	*Klebsiella* phage KpV475	100	4e-40	100	YP_009280697.1
ORF41	+	22702	23124	140	Endonuclease VII	*Klebsiella* phage Kp2	100	2e-96	100	YP_009188344.1
ORF42	+	23121	23615	164	Polynucleotide kinase	*Klebsiella* phage vB_KpnP_SU552A	100	4e-116	99.39	YP_009204821.1
ORF43	+	23612	23926	104	Hypothetical protein	*Klebsiella* phage vB_KpnP_SU552A	100	2e-69	100	YP_009204822.1

ORF44	+	23937	24029	30	Hypothetical protein	*Klebsiella* phage vB_KpnP_IME308	100	8e-13	100	QEQ50366.1
ORF45	+	24068	26536	822	RNA polymerase	*Klebsiella* virus KpV2883	100	0.0	99.03	QMP82077.1
ORF46	+	26560	27000	146	Hypothetical protein	*Klebsiella* virus KpV2883	100	5e-102	98.63	QMP82078.1
ORF47	+	26997	27260	87	Hypothetical protein	*Klebsiella* phage KP34	100	1e-50	100	YP_003347631.1
ORF48	+	27270	28865	531	Head-tail connector protein	*Klebsiella* virus KpV2883	100	0.0	99.62	QMP82080.1
ORF49	+	28880	29722	280	Head scaffolding protein	*Klebsiella* phage vB_KpnP_SU552A	100	0.0	99.64	YP_009204827.1
ORF50	+	29748	30767	339	Capsid protein	*Klebsiella* virus KpV2883	100	0.0	98.23	QMP82082.1
ORF51	+	30779	30961	60	Hypothetical protein	*Klebsiella* phage BUCT86	100	2e-30	98.33	UFX84353.1
ORF52	+	31050	31610	186	Tail protein	*Klebsiella* phage myPSH1235	100	4e-133	98.92	YP_009799485.1
ORF53	+	31620	33980	786	Non-contractile tail tubular protein	*Klebsiella* phage 6995	100	0.0	98.60	URY99708.1
ORF54	+	33982	34569	195	Internal virion protein	*Klebsiella* phage vB_KpnP_SU552A	100	9e-136	99.49	YP_009204832.1
ORF55	+	34587	37271	894	Hypothetical protein	*Klebsiella* phage VLC3	100	0.0	98.66	QGZ00873.1
ORF56	+	37322	41020	1232	Tail fiber protein	*Caudoviricetes* sp.	100	0.0	96.10	DAL65533.1
ORF57	+	41022	43418	798	Depolymerase	*Klebsiella* virus KpV2883	100	0.0	94.49	QMP82089.1

Supplemental Table S3. Annotated ORFs in the genome of vB_KpnS_SCNJ1-C

ORF	Strand	Start	Stop	Length (AA)	Putative function	Best-match BLASTp result	Query cover (%)	E-values	Identity (%)	Accession
ORF1	+	12	398	128	Lysozyme	*Caudoviricetes* sp.	100%	2e-90	99.22	DAE85906.1
ORF2	+	395	640	81	Hypothetical protein	*Klebsiella pneumoniae*	100%	5e-51	100.00%	WP_142483241.1
ORF3	+	615	1109	164	Rz-like spanin	*Klebsiella* phage KP591P1	100%	2e-110	98.17%	YP_010685383.1
ORF4	-	1268	1140	42	Hypothetical protein	*Klebsiella* phage BUCT541	100%	1e-19	92.86%	YP_010685956.1
ORF5	-	1489	1265	74	Hypothetical protein	*Siphoviridae* sp. cthqG28	100%	9e-46	98.65%	DAD75123.1
ORF6	-	1752	1489	87	Hypothetical protein	*Klebsiella pneumoniae*	100%	2e-50	89.66%	WP_142483238.1
ORF7	-	2216	1749	155	Hypothetical protein	*Caudoviricetes* sp.	100%	3e-97	90.97%	DAF74722.1
ORF8	-	2410	2216	64	Hypothetical protein	*Klebsiella* phage vB_KpnS_MK54	100%	7e-37	95.31%	YP_010685264.1
ORF9	-	2610	2410	66	Hypothetical protein	*Klebsiella pneumoniae*	100%	1e-39	98.48%	HBU8799217.1
ORF10	+	2706	2921	71	Hypothetical protein	*Escherichia coli*	100%	5e-43	98.59%	WP_236516193.1
ORF11	+	3005	3196	63	Hypothetical protein	*Caudoviricetes* sp.	100%	2e-35	98.41%	DAN88399.1
ORF12	+	3193	3417	74	Hypothetical protein	*Caudoviricetes* sp.	100%	3e-41	93.24%	DAF74716.1
ORF13	+	3414	3650	78	Lar-like restriction alleviation protein	*Klebsiella* phage ZCKP8	100%	2e-40	78.21%	YP_010685615.1
ORF14	+	3640	4110	156	MarR family transcrition regulator	*Klebsiella* phage VLCpiS13f	100%	6e-111	98.08%	YP_010685130.1
ORF15	+	4279	4530	83	Hypothetical protein	*Salmonella* enterica subsp. enterica serovar Reading	97%	1e-29	68.67%	MCO9872112.1

ORF16	+	4527	4871	114	Hypothetical protein	*Caudoviricetes* sp.	100%	4e-76	96.49%	DAF74711.1
ORF17	+	5054	6454	466	Prohead core protein serine protease	*Caudoviricetes* sp.	100%	0.0	99.79%	DAV69956.1
ORF18	+	6454	6942	162	Head decoration	*Klebsiella* phage VLCpiS13e	100%	8e-100	91.98%	YP_010685251.1
ORF19	+	6954	8042	362	Major head protein	*Caudoviricetes* sp.	100%	0.0	98.34%	DAP27990.1
ORF20	+	8087	8461	124	Hypothetical protein	*Salmonella enterica*	100%	1e-72	82.26%	EGT9551703.1
ORF21	+	8454	8648	64	Hypothetical protein	*Caudoviricetes* sp.	100%	1e-36	98.44%	DAR02999.1
ORF22	+	8691	9509	272	Hypothetical protein	*Klebsiella* phage vB_KpnS_MK54	100%	0.0	97.79%	YP_010685276.1
ORF23	-	9817	9539	92	Restriction alleviation protein	*Caudoviricetes* sp.	98%	4e-30	59.34	DAE43019.1
ORF24	-	10017	9844	57	Hypothetical protein	*Klebsiella* phage BUCT610	100%	2e-34	100.00%	YP_010685848.1
ORF25	-	10271	10014	85	Hypothetical protein	*Caudoviricetes* sp.	100%	6e-54	97.65%	DAO12166.1
ORF26	-	11086	10271	271	ParB-like partition	*Klebsiella* phage YX3973	100%	0.0	99.63%	YP_010054409.1
ORF27	-	11210	11094	38	Hypothetical protein	*Klebsiella* phage vB_KpnS_ZX4	100%	3e-16	97.37%	YP_010054525.1
ORF28	-	11470	11207	87	Anti-restriction protein	*Klebsiella* phage VLCpiS13f	100%	8e-51	94.25%	YP_010685117.1
ORF29	+	11701	11988	95	Membrane protein	*Klebsiella* phage YX3973	100%	8e-62	100.00%	YP_010054411.1
ORF30	+	11966	12358	130	Hypothetical protein	*Klebsiella* phage YX3973	100%	4e-89	98.46%	YP_010054412.1
ORF31	+	12355	12489	44	Hypothetical protein	*Klebsiella* phage KP591P1	100%	5e-21	97.73%	YP_010685408.1
ORF32	+	12477	12821	114	Hypothetical protein	*Escherichia coli*	100%	3e-78	99.12%	WP_236516175.1
ORF33	+	12825	13241	138	Minor capsid protein	*Caudoviricetes* sp.	100%	9e-94	98.55%	DAN88321.1
ORF34	+	13238	13621	127	Tail completion protein	*Caudoviricetes* sp.	100%	2e-88	100.00%	DAF74636.1
ORF35	-	13698	13622	-	tRNA-Met-CAT	-	-	-	-	-
ORF36	-	13779	13702	-	Trna-Arg-TCT	-	-	-	-	-
ORF37	+	14235	14990	251	Major tail protein	*Caudoviricetes* sp.	100%	6e-169	94.02%	DAP27996.1
ORF38	-	15175	15068	35	Hypothetical protein	Not hit	-	-	-	-
ORF39	-	15453	15187	88	Hypothetical protein	*Caudoviricetes* sp.	95%	1e-50	90.48%	DAH53619.1
ORF40	-	15664	15467	65	Hypothetical protein	Not hit	-	--	-	-
ORF41	-	16011	15664	115	Hypothetical protein	*Klebsiella* phage VLCpiS13d	98%	1e-49	73.45%	YP_010684971.1
ORF42	-	16142	16008	44	Hypothetical protein	*Klebsiella* phage VLCpiS13d	100%	1e-21	100.00%	YP_010684972.1
ORF43	+	16240	16947	235	Hypothetical protein	*Klebsiella* phage vB_KpnS_MK54	100%	9e-173	99.57%	YP_010685293.1
ORF44	+	16947	20237	1096	Tail length tape measure protein	*Caudoviricetes* sp.	100%	0.0	95.26%	DAR02950.1
ORF45	+	20237	20710	157	Hypothetical protein	*Caudoviricetes* sp.	100%	1e-109	95.54%	DAZ46274.1
ORF46	-	21198	20707	163	endonuclease	*Caudoviricetes* sp.	100%	4e-116	99.39%	DAZ46224.1
ORF47	+	21378	21848	156	Minor tail protein	*Klebsiella* phage BUCT610	100%	2e-109	98.72%	YP_010685869.1
ORF48	+	21811	22269	152	Type VI secretion exported 1	*Caudoviricetes* sp.	100%	1e-107	98.03%	DAV90930.1
ORF49	+	22217	24697	826	Tail protein	*Caudoviricetes* sp.	100%	0.0	98.67%	DAN88317.1
ORF50	+	24736	26820	694	Depolymerase	*Klebsiella* phage vB_KpnS_MK54	100%	0.0	97.69%	YP_010685299.1
ORF51	-	27352	26849	167	Single-stranded DNA-	*Klebsiella* phage VLCpiS13f	100%	3e-116	97.60%	YP_010685179.1

					binding protein					
ORF52	-	28021	27362	219	Recombinase	*Klebsiella* phage VLCpiS13f	100%	4e-157	99.54%	YP_010685178.1
ORF53	-	29001	28045	318	Exonuclease VIII	*Vibrio* phage pYD38-A	100%	0.0	97.17%	YP_008126224.1
ORF54	-	29570	29004	188	Homing endonuclease	*Caudoviricetes* sp.	100%	3e-109	80.85%	DAV90926.1
ORF55	-	30101	29676	141	Hypothetical protein	*Klebsiella* phage VLCpiS13e	100%	2e-100	98.58%	YP_010685206.1
ORF56	-	30622	30098	174	Homing endonuclease	*Caudoviricetes* sp.	100%	6e-123	97.70%	DAN88328.1
ORF57	-	32540	30609	643	DEAD/DEAH box helicase family protein	*Klebsiella* phage BUCT541	98%	0.0	98.90%	YP_010685907.1
ORF58	+	32615	33508	297	Primase helicase	*Caudoviricetes* sp.	100%	0.0	96.97%	DAF78393.1
ORF59	-	33738	33523	71	Hypothetical protein	*Klebsiella* phage VLCpiS13f	100%	1e-44	98.59%	YP_010685171.1
ORF60	-	35225	33735	496	Hypothetical protein	Flyfo *siphovirus* Tbat1_6	100%	0.0	99.80%	YP_010685020.1
ORF61	-	35437	35222	71	Hypothetical protein	*Caudoviricetes* sp.	100%	5e-44	100.00%	DAF74679.1
ORF62	+	35939	36154	71	Hypothetical protein	*Klebsiella* phage VLCpiS13a	100%	3e-39	90.14%	YP_010685778.1
ORF63	+	36151	36336	61	Hypothetical protein	*Klebsiella* virus KpV2811	100%	3e-36	98.36%	YP_010054493.1
ORF64	+	36352	36960	202	Domain-containing protein	*Klebsiella pneumoniae*	63%	8e-78	88.55%	RYI79752.1
ORF65	+	36957	37049	30	Hypothetical protein	*Caudoviricetes* sp	100%	2e-09	93.33%	DAN88356.1
ORF66	+	37053	37211	52	Hypothetical protein	Bacteriophage sp.	100%	8e-30	98.08%	UVM90469.1
ORF67	+	37211	37405	64	Hypothetical protein	*Caudoviricetes* sp.	100%	2e-35	89.06%	DAZ46237.1
ORF68	+	37486	37929	147	Hypothetical protein	*Caudoviricetes* sp.	100%	4e-103	100.00%	DAE85929.1
ORF69	+	38067	39491	474	Terminase large subunit	*Klebsiella* phage vB_Kpn_ZC2	100%	0.0	98.31%	YP_010685671.1
ORF70	-	40088	39798	96	Polynucleotide kinase	*Klebsiella* phage BUCT610	100%	2e-62	100.00%	YP_010685813.1
ORF71	+	40243	40416	57	Hypothetical protein	*Klebsiella* phage VLCpiS13e	100%	1e-31	98.25%	YP_010685225.1
ORF72	+	40413	40580	55	Hypothetical protein	*Caudoviricetes* sp.	100%	5e-20	74.55%	DAV89248.1
ORF73	+	40580	40876	98	Hypothetical protein	*Klebsiella* phage VLCpiS13f	100%	5e-58	90.82%	YP_010685153.1
ORF74	+	40873	41070	65	Hypothetical protein	*Klebsiella pneumoniae*	100%	2e-38	96.92%	WP_142483250.1
ORF75	+	41067	41270	67	Hypothetical protein	*Klebsiella* phage KP591P1	98%	7e-37	93.94%	YP_010685374.1
ORF76	+	41281	41463	60	Hypothetical protein	Flyfo *siphovirus* Tbat1_6	100%	5e-34	95.00%	YP_010685045.1
ORF77	+	41680	43128	482	Portal protein	*Klebsiella* virus KpV2811	100%	0.0	96.06%	YP_010054478.1
ORF78	+	43109	44056	315	Hypothetical protein	*Klebsiella pneumoniae*	98%	0.0	95.50%	HBU8799203.1
ORF79	+	44146	45045	299	GIY-YIG nuclease family protein	*Salmonella enterica*	100%	0.0	80.94%	WP_249979768.1
ORF80	-	45299	45099	66	Hypothetical protein	*Salmonella* phage LPST10	100%	7e-179	79.26%	YP_010053829.1
ORF81	-	45417	45277	46	Hypothetical protein	*Caudoviricetes* sp.	100%	4e-22	93.48%	DAZ46242.1
ORF82	-	45707	45417	96	Hypothetical protein	*Klebsiella* virus KpV2811	100%	6e-58	95.83%	YP_010054472.1
ORF83	+	45806	45997	63	endolysin	*Klebsiella* phage VLCpiS13c	95%	6e-36	100.00%	YP_010686152.1

Supplemental Table S4. Annotated ORFs in the genome of vB_KpnM_SCNJ1-Y

ORF	Strand	Start	Stop	Length (AA)	Putative function	Best-match BLASTp result	Query cover (%)	E-values	Identity (%)	Accession
ORF1	+	1	420	139	Terminase small subunit	*Klebsiell*a phage VLCpiM12a	100%	2e-93	98.56%	YP_010683877.1
ORF2	+	413	1798	461	Terminase large subunit	*Klebsiella* phage vB_KpnM_KpV52	100%	0.0	98.92%	YP_009597530.1
ORF3	-	2668	1805	287	Endonuclease	*Klebsiella* phage BUCT_49532	100%	0.0	96.52%	YP_010683735.1
ORF4	+	2727	4145	472	Portal protein	*Klebsiella* phage Geezett	100%	0.0	99.15%	YP_010683888.1
ORF5	-	4463	4158	101	Hypothetical protein	*Staphylococcus haemolyticus*	100%	9e-68	99.01%	WP_227946425.1
ORF6	-	4872	4468	134	HNH endonuclease	Bacteriophage sp.	98%	8e-55	66.67%	UVM95876.1
ORF7	+	5105	5902	265	Minor capsid protein	*Escherichia* phage ZCEC13	100%	2e-137	72.83%	UPU16094.1
ORF8	-	6611	5892	239	GIY-YIG nuclease family protein	*Enterovibrio paralichthyis*	37%	8e-05	33.33%	WP_218357777.1
ORF9	-	6793	6611	60	Hypothetical protein	*Caudoviricetes* sp.	100%	2e-26	75.00%	DAE41567.1
ORF10	-	6987	6790	65	Outer membrane adhesin like protein	*Klebsiella* phage vB_KpnM_IME346	100%	2e-33	87.69%	YP_010684263.1
ORF11	-	7133	6984	49	Glycosyltransferase	*Klebsiella* phage VLCpiM12a	100%	2e-25	93.88%	YP_010683871.1
ORF12	-	7357	7130	75	Nucleotide kinase	*Klebsiella* phage Geezett	100%	1e-44	93.33%	YP_010683899.1
ORF13	+	7432	8520	362	Head maturation protease	*Klebsiella* phage 1611E-K2-1	100%	0.0	98.63%	YP_010684684.1
ORF14	+	8532	9020	162	Virion structural protein	*Klebsiella* phage VLCpiM12a	100%	1e-102	98.15%	YP_010683864.1
ORF15	+	9023	10057	344	Major capsid protein	*Caudoviricetes* sp.	100%	0.0	100.00%	DAK72439.1
ORF16	+	10154	11734	526	Endonuclease-like protein	*Klebsiella* phage 1611E-K2-1	100%	0.0	95.82%	YP_010684681.1
ORF17	+	11768	12085	105	Recombination endonuclease VII	*Caudoviricetes* sp.	100%	7e-68	97.14%	DAR38700.1
ORF18	+	12104	12688	194	Hypothetical protein	*Klebsiella* phage vB_KleM_KB2	100%	8e-133	97.94%	YP_010684898.1
ORF19	+	12685	13041	118	Hypothetical protein	*Klebsiella* phage BUCT_49532	100%	2e-76	96.61%	YP_010683673.1
ORF20	+	13043	13462	139	Head to tail adaptor	*Caudoviricetes* sp.	100%	7e-99	98.56%	DAR38668.1
ORF21	-	13609	13478	43	Hypothetical protein	*Klebsiella* phage Geezett	100%	1e-20	93.02%	YP_010683907.1
ORF22	-	13905	13606	99	Hypothetical protein	*Caudoviricetes* sp.	100%	8e-67	100.00%	DAF30618.1
ORF23	+	14313	14816	167	Tail completion	*Klebsiella* phage pKp383	100%	3e-113	95.81%	YP_010684204.1
ORF24	+	14813	15166	117	Minor capsid protein	*Caudoviricetes* sp.	100%	3e-79	97.44%	DAK85296.1
ORF25	+	15153	15734	193	Tail-completion protein	*Klebsiella* phage 1611E-K2-1	100%	2e-140	98.96%	YP_010684670.1
ORF26	+	15775	16911	378	Tail sheath	*Klebsiella* phage 1611E-K2-1	100%	0.0	98.94%	YP_010684669.1
ORF27	-	17813	16926	295	Endonuclease	*Edwardsiella* phage PEi21	97%	1e-52	38.10%	YP_008869216.1
ORF28	+	17850	18266	138	Virion structural protein	*Klebsiella* phage vB_KpnM_FZ14	100%	3e-92	98.55%	YP_010684829.1
ORF29	-	18835	18293	180	Domain-containing protein	*Escherichia* phage ZCEC13	61%	8e-61	84.68%	YP_010684609.1
ORF30	-	19116	18832	94	Hypothetical protein	*Klebsiella* phage vB_KpnM_IME346	100%	9e-62	96.81%	YP_010684238.1
ORF31	+	19234	19608	124	Tail assembly chaperone protein	*Caudoviricetes* sp.	100%	4e-84	97.58%	DAK83421.1

ORF32	+	19795	20091	98	Virion structural protein	*Klebsiella* phage SBP	100%	3e-65	98.98%	YP_010684502.1
ORF33	+	20091	21563	490	Tail length tape measure protein	*Klebsiella* phage vB_KleM_KB2	100%	0.0	95.92%	YP_010684921.1
ORF34	+	21566	22990	474	Tail protein	*Caudoviricetes* sp.	100%	0.0	97.89%	DAI72789.1
ORF35	+	22987	23637	216	Tail fiber protein	*Klebsiella* phage Geezett	100%	2e-152	99.54%	YP_010683923.1
ORF36	+	23664	23822	52	Hypothetical protein	*Caudoviricetes* sp.	42%	7e-06	95.45%	DAR38752.1
ORF37	+	24214	24888	224	Baseplate spike	*Klebsiella* phage vB_KleM_KB2	100%	4e-164	100.00%	YP_010684833.1
ORF38	+	24888	25235	115	Baseplate wedge subunit	*Klebsiella* phage JD001	100%	4e-76	100.00%	YP_007392889.1
ORF39	+	25228	26436	402	Baseplate wedge subunit	*Klebsiella* phage vB_KpnM_KpV79	100%	0.0	97.26%	YP_009615312.1
ORF40	+	26433	28508	691	Depolymerase	*Klebsiella* phage vB_KpnS_MK54	83%	0.0	90.99%	YP_010685299.1
ORF41	+	28660	29373	237	Structural protein	*Klebsiella* phage vB_KpnM_KpV52	100%	1e-166	94.94%	YP_009597572.1
ORF42	+	29377	30270	297	Tail-fiber protein	*Klebsiella* phage vB_KpnM_IME346	100%	0.0	96.63%	YP_010684224.1
ORF43	-	30501	30265	78	Hypothetical protein	*Klebsiella pneumoniae*	100%	8e-49	98.72%	RZM50411.1
ORF44	-	30827	30498	109	Rz-like spanin	*Klebsiella* phage 1611E-K2-1	100%	5e-66	92.66%	YP_010684735.1
ORF45	-	31384	30824	186	Endolysin	*Klebsiella* phage vB_KpnM_KpV52	100%	4e-134	98.92%	YP_009597576.1
ORF46		31439	31549	36	Hypothetical protein	*Klebsiella* phage pKp383	100%	2e-14	100.00%	YP_010684158.1
ORF47	+	31553	31963	136	Hypothetical protein	*Caudoviricetes* sp.	100%	6e-91	97.06%	QHJ80458.1
ORF48	+	31990	32271	93	Hypothetical protein	*Caudoviricetes* sp.	100%	2e-58	98.92%	QHJ80459.1
ORF49	-	32629	32288	113	Domain-containing protein	*Klebsiella* phage vB_KpnM_FZ14	100%	1e-76	98.23%	YP_010684774.1
ORF50	-	33219	32626	197	DNA polymerase	*Klebsiella* phage vB_KpnM_JustaPhage	100%	8e-140	95.43%	YP_010684051.1
ORF51	-	34099	33209	296	Hypothetical protein	*Klebsiella* phage vB_KpnM_15-38_KLPPOU148	100%	0.0	97.97%	YP_010683594.1
ORF52	-	35619	34096	507	DNA polymerase	*Caudoviricetes* sp.	100%	0.0	97.04%	DAI64904.1
ORF53	-	35817	35650	55	Domain-containing protein	*Klebsiella* phage vB_KleM_KB2	100%	2e-30	98.18%	YP_010684848.1
ORF54	-	35936	35805	43	Hypothetical protein	*Klebsiella* phage BUCT_47333	100%	2e-20	97.67%	YP_010683783.1
ORF55	-	36788	35985	267	ssDNA binding protein	*Klebsiella* phage VLCpiM12a	100%	0.0	98.15%	YP_010683824.1
ORF56	-	37005	36874	43	Hypothetical protein	*Klebsiella* phage BUCT_49532	100%	3e-31	100.00%	YP_010683709.1
ORF57	-	38156	37002	384	Exonuclease	*Klebsiella* phage vB_KleM_KB2	97%	0.0	96.78%	YP_010684852.1
ORF58	-	38280	38119	53	Hypothetical protein	*Klebsiella* phage 05F01	88%	2e-04	48.94%	BBK09221.1
ORF59	-	39193	38285	302	Hypothetical protein	*Klebsiella* phage pKp383	100%	4e-151	85.43%	YP_010684167.1
ORF60	+	39334	39552	72	Methyltransferase domain	*Klebsiella* phage vB_KpnM_FZ14	100%	8e-44	97.22%	YP_010684783.1
ORF61	+	39556	39753	65	Hypothetical protein	*Klebsiella* phage vB_KpnM_FZ14	100%	2e-40	98.46%	YP_010684784.1
ORF62	+	39762	39932	56	Hypothetical protein	Phage vB_KsaM-C1	100%	6e-31	96.43%	UTC25978.1
ORF63	+	39935	40165	76	Hypothetical protein	*Pantoea septica*	85%	9e-07	40.00%	WP_277268291.1

ORF64	+	40178	41821	547	DNA helicase	*Klebsiella* phage vB_KpnM_IME346	99%	0.0	97.99%	YP_010684282.1
ORF65	+	41818	42246	142	NADAR protein	*Caudoviricetes* sp.	100%	9e-99	97.89%	DAF30604.1
ORF66	+	42245	42716	157	Nuclease	*Caudoviricetes* sp.	100%	2e-110	98.09%	DAR38684.1
ORF67	+	42716	43048	110	Hypothetical protein	*Klebsiella pneumoniae* subsp. pneumon	97%	6e-41	62.28%	HBQ5946563.1
ORF68	+	43103	43381	92	TetR family transcriptional regulator	*Klebsiella pneumoniae*	100%	2e-55	95.65%	WP_130243603.1
ORF69	+	43404	45761	785	dsDNA helicase	*Caudoviricetes* sp.	99%	0.0	97.83%	DAI64906.1
ORF70	+	45857	46009	50	Hypothetical protein	*Escherichia coli*	100%	2e-26	100.00%	WP_236502019.1
ORF71	+	45996	46277	93	Hypothetical protein	*Klebsiella* phage BUCT_47333	100%	3e-54	87.10%	YP_010683788.1
ORF72	+	46382	46495	37	Anticodon nuclease activator family	*Escherichia* phage ZCEC13	100%	3e-13	81.08%	UPU16113.1
ORF73	+	46540	46713	57	Hypothetical protein	*Klebsiella* phage VLCpiM12a	100%	3e-33	100.00%	YP_010683809.1
ORF74	+	46701	47105	134	Hypothetical protein	*Klebsiella pneumoniae*	83%	4e-66	88.39%	MCE0146380.1
ORF75	+	47102	47401	99	Polynucleotide kinase	*Klebsiella* phage Geezett	100%	8e-66	98.99%	YP_010683959.1
ORF76	+	47394	47684	96	Hypothetical protein	Bacteriophage sp.	100%	1e-62	97.92%	QHJ81679.1
ORF77	+	47684	48826	380	Domain-containing protein	*Klebsiella* phage 1611E-K2-1	100%	0.0	97.63%	YP_010684704.1
ORF78	+	48813	49175	120	HNH endonuclease	*Klebsiella* phage BUCT_49532	100%	2e-73	74.68%	YP_010683729.1
ORF79	+	49172	49333	53	Hypothetical protein	Bacteriophage sp.	100%	2e-27	94.34%	QHJ82135.1
ORF80	-	49951	49370	193	Hypothetical protein	*Klebsiella* phage Geezett	100%	1e-135	94.30%	YP_010683962.1

Supplemental Table S5. Optimal MOI for phages

MOI(PFU/CFU)	Phage (PFU/mL)	Bacteria (CFU/mL)	Progeny of phage Z (PFU/mL)	Progeny of phage C (PFU/mL)	Progeny of phage Y (PFU/mL)
100	10^{10}	10^{8}	2.67×10^{9}	2.6×10^{9}	5.26×10^{8}
10	10^{9}	10^{8}	1.2×10^{9}	7.6×10^{8}	3.67×10^{8}
1	10^{8}	10^{8}	6.67×10^{8}	1.4×10^{9}	7.33×10^{8}
0.1	10^{7}	10^{8}	4×10^{8}	3.9×10^{9}	1.50×10^{9}
0.01	10^{6}	10^{8}	1.27×10^{9}	1.02×10^{10}	3.36×10^{9}
0.001	10^{5}	10^{8}	2.93×10^{9}	1.68×10^{10}	6.0×10^{9}
0.0001	10^{4}	10^{8}	3.6×10^{9}	4.3×10^{10}	8.2×10^{9}

phage Z represents vB_KpnA_SCNJ1-Z; phage C represents vB_KpnS_SCNJ1-C; phage Y represents vB_KpnM_SCNJ1-Y.

Supplemental Table S6. Statistical analysis within phage treatment groups in the *in vitro* experiment at MOI 1

Phage	vB_KpnA_SCNJ1-Z	vB_KpnS_SCNJ1-C	vB_KpnM_SCNJ1-Y	cocktail
vB_KpnA_SCNJ1-Z	/	ns	ns	ns
vB_KpnS_SCNJ1-C	ns	/	ns	ns
vB_KpnM_SCNJ1-Y	ns	ns	/	ns
cocktail	ns	ns	ns	/

ns represents not significant

Supplemental Table S7. Statistical analysis within phage treatment groups in the *in vitro* experiment at MOI 10

Phage	vB_KpnA_SCNJ1-Z	vB_KpnS_SCNJ1-C	vB_KpnM_SCNJ1-Y	cocktail
vB_KpnA_SCNJ1-Z	/	ns	ns	ns
vB_KpnS_SCNJ1-C	ns	/	ns	ns
vB_KpnM_SCNJ1-Y	ns	ns	/	ns
cocktail	ns	ns	ns	/

ns represents not significant

Supplemental Table S8 Statistical analysis within phage treatment groups in the *in vitro* experiment at MOI 100

Phage	vB_KpnA_SCNJ1-Z	vB_KpnS_SCNJ1-C	vB_KpnM_SCNJ1-Y	cocktail
vB_KpnA_SCNJ1-Z	/	ns	*	ns
vB_KpnS_SCNJ1-C	ns	/	ns	ns
vB_KpnM_SCNJ1-Y	*	ns	/	**
cocktail	ns	ns	**	/

ns represents not significant, * represents $p<0.05$, ** represents $p<0.01$

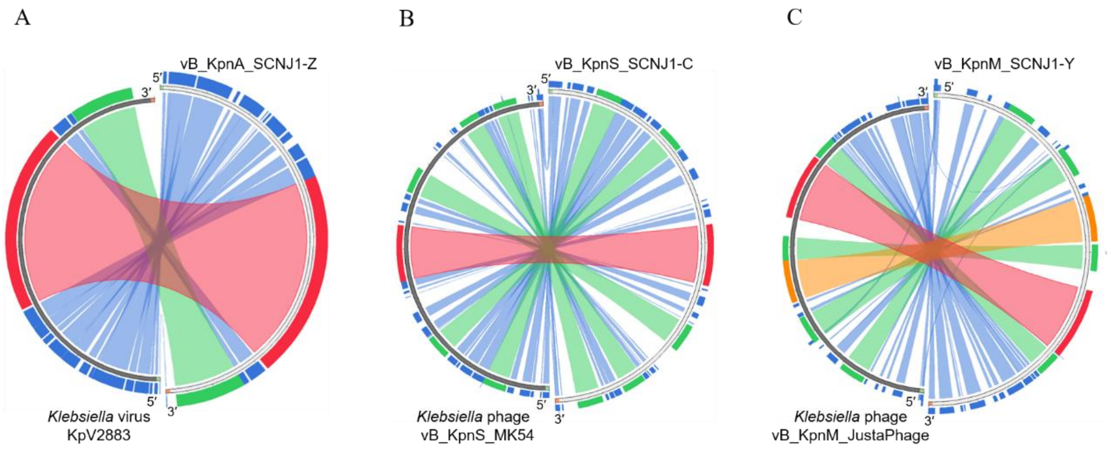

Supplemental Figure 1. Circos plot depicting sequence similarities of the three phages. (A) vB_KpnA_SCNJ1-Z with *Klebsiella* virus KpV2883, (B) vB_KpnS_SCNJ1-C with *Klebsiella* phage vB_KpnS_MK54, (C) vB_KpnM_SCNJ1-Y with *Klebsiella* phage vB_KpnM_JustaPhage. The red color signifies a high sequence similarity followed by orange, green, and blue. Ratio coloring with blue ⩽ 0.25, green ⩽ 0.50, orange ⩽ 0.75, and red > 0.75.

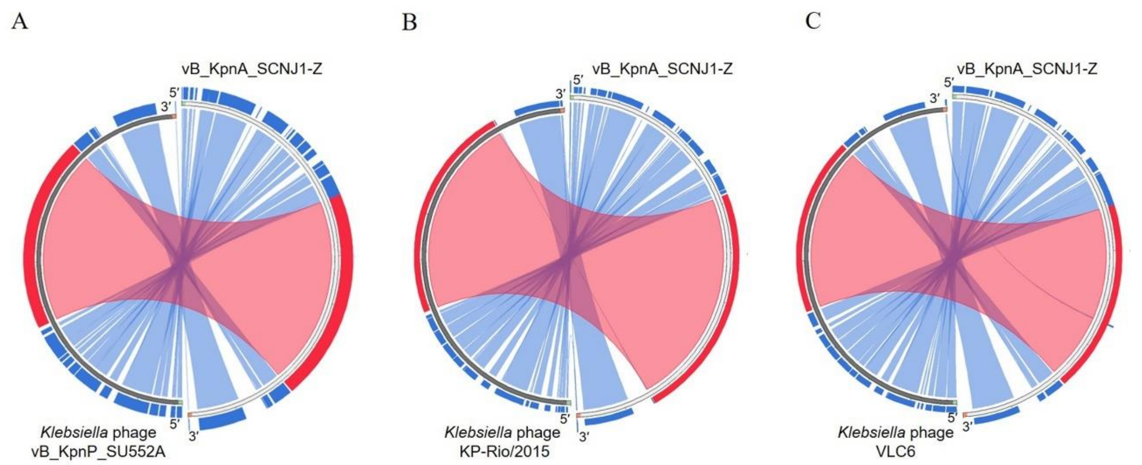

Supplemental Figure 2. Circos plot illustrating the sequence similarities of *Klebsiella* phage vB_KpnA_SCNJ1-Z with (A) *Klebsiella* phage vB_KpnP_SU552A, (B) *Klebsiella* phage KP-Rio/2015, and (C) *Klebsiella* phage VLC6. The degree of sequence similarity is depicted by colors ranging from blue (≤ 0.25) to green (≤ 0.50), orange (≤ 0.75), and red (> 0.75).

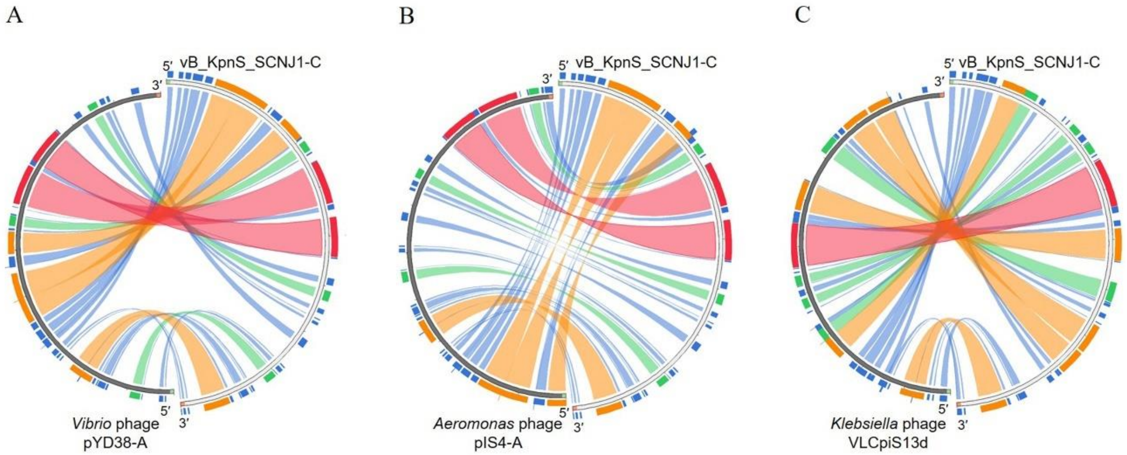

Supplemental Figure 3. Circos plot illustrating the sequence similarities of *Klebsiella* phage vB_KpnS_SCNJ1-C with (A) *Vibrio* phage pYD38-A, (B) *Aeromonas* phage pIS4-A, and (C) *Klebsiella* phage VLCpiS13d. The degree of sequence similarity is depicted by colors ranging from blue (≤ 0.25) to green (≤ 0.50), orange (≤ 0.75), and red (> 0.75).

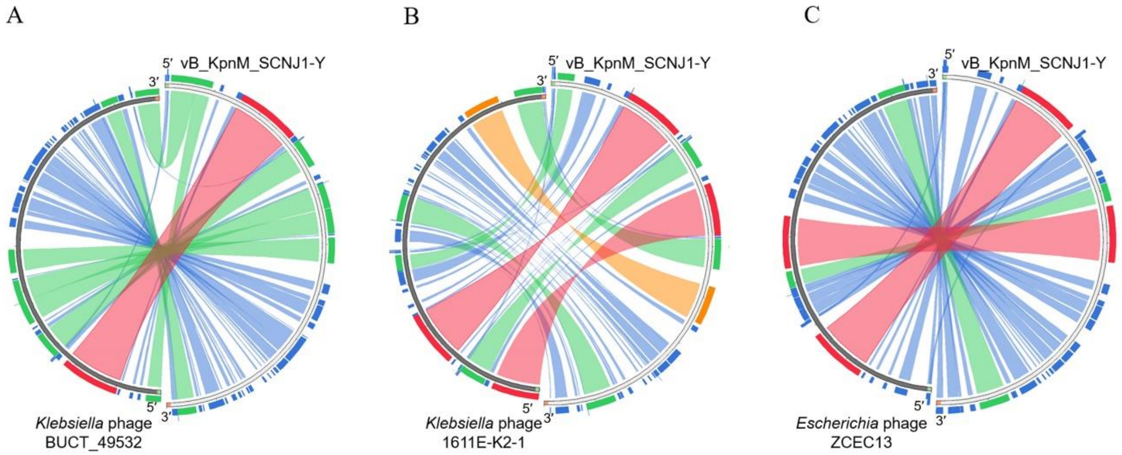

Supplemental Figure 4. Circos plot illustrating the sequence similarities of *Klebsiella* phage vB_KpnM_SCNJ1-Y with (A) *Klebsiella* phage BUCT_49532, (B) *Klebsiella* phage 1611E-K2-1, and (C) *Escherichia* phage ZCEC13. The degree of sequence similarity is depicted by colors ranging from blue (≤ 0.25) to green (≤ 0.50), orange (≤ 0.75), and red (> 0.75).
